# Preparation and performance evaluation of a novel orthodontic adhesive incorporating composite dimethylaminohexadecyl methacrylate—Polycaprolactone fibers

**DOI:** 10.1371/journal.pone.0304143

**Published:** 2024-05-23

**Authors:** Xuecheng Xu, Qihan Yuan, Linlin Xu, Mingchang Hu, Jidong Xu, Yuanfei Wang, Yu Song

**Affiliations:** 1 School of Stomatology, Qingdao University, Qingdao, China; 2 Department of Central Laboratory, Qingdao Stomatological Hospital Affiliated to Qingdao University, Qingdao, China; 3 Department of Orthodontics, Qingdao Stomatological Hospital Affiliated to Qingdao University, Qingdao, China; University of Sharjah, UNITED ARAB EMIRATES

## Abstract

This study addressed enamel demineralization, a common complication in fixed orthodontic treatment, by evaluating a novel orthodontic adhesive with DMAHDM-PCL composite fibers. These fibers, produced through electrospinning, were incorporated into orthodontic adhesive to create experimental formulations at different concentrations and a control group. The study assessed antimicrobial properties, biosafety, and mechanical characteristics. New orthodontic adhesive exhibited significant bacteriostatic effects, reducing bacterial biofilm activity and concentrations. Incorporating 1% and 3% DMAHDM-PCL did not affect cytocompatibility. Animal tests confirmed no inflammatory irritation. Shear bond strength and adhesive residual index results indicated that antimicrobial fibers didn’t impact bonding ability. In conclusion, orthodontic adhesives with 3% DMAHDM-PCL fibers are potential antimicrobial bonding materials, offering a comprehensive solution to enamel demineralization in orthodontic patients.

## 1 Introduction

Fixed orthodontic treatment, a widely utilized method in orthodontics, is favored for its effectiveness and precision [1, 2]. However, wearing fixed orthodontic appliances can alter the oral environment, leading to plaque accumulation around the brackets, which is challenging to clean. Bacterial acid production can result in the formation of chalky, opaque spots or plaques on the enamel surface, clinically referred to as "white spot lesions" (WSL) [3, 4]. WSL is the most common complication associated with fixed orthodontic treatment, compromising both the aesthetic and health aspects of orthodontic care [5]. *Streptococcus mutans* is the predominant causative agent of WSL [6]. The prevalence of WSL varies significantly, with reported incidence rates ranging from 2% to 97% [7].

Prevention of enamel demineralization relies on patients maintaining good oral hygiene conditions. However, the antimicrobial efficacy of methods dependent on patient compliance is often unsatisfactory [8, 9]. In recent years, efforts have been made to develop antimicrobial orthodontic adhesives, which are materials capable of killing bacteria and reducing biofilm formation independently of patient compliance. Quaternary ammonium salts (QAS) function as bactericidal antimicrobial agents by disrupting the electrical equilibrium of the cell membrane, leading to its disruption, destruction of bacterial proteins and enzymes, and denaturation of the bacteria [10–14]. It has been observed that adding 5% dimethylaminohexadecyl methacrylate (DMAHDM) to commercial orthodontic adhesives through physical mixing exhibits the most robust anti-biofilm efficacy without compromising bond strength [15]. Based on previous studies, DMAHDM was synthesized using a modified Menschutkin reaction method using tertiary amine groups reacted with organic halides [15, 16]. However, few studies have investigated the potential toxic effects on cells or whether these materials irritate the oral mucosa when applied in the oral environment [17, 18]. Furthermore, traditional methods of physical mixing may lead to drug accumulation and fail to achieve uniform distribution within commercial orthodontic adhesives. Micro/nanomaterials have gained widespread use in recent years across various disciplines. Electrospinning (ES) is a well-established technique for fabricating scaffolds composed of micro/nanofibers, which find extensive application in biomedicine due to their large surface area-to-volume ratios, high porosity, and small pore sizes [19–21]. Polycaprolactone (PCL) is commonly employed as a medical biodegradable material and in drug-controlled release systems for tissue engineering, owing to its favorable biodegradability [22], biocompatibility [23], and non-toxicity. Thus, by harnessing the advantages of these carriers and materials, it becomes possible to increase the contact area of drug-carrying materials, reduce drug accumulation, and overcome the "contact inhibition" antimicrobial characteristics.

In this study, for the first time, DMAHDM was prepared into composite nanofibers using electrostatic spinning technology and then incorporated into commercial orthodontic adhesives to synthesize a novel orthodontic bonding agent with excellent biosafety and long-term antimicrobial properties. The novel adhesive’s shear bond strength and antimicrobial properties were evaluated with particular emphasis on its biosafety. The amounts of DMAHDM-PCL fibers to be added within the biosafety range were clarified, providing a new approach for enamel protection in fixed orthodontic treatment.

## 2 Materials and methods

Participant privacy and confidentiality were carefully protected throughout the planning, execution, and reporting process. Informed consent was obtained from the patients for the acquisition of isolated teeth. The experimental animal testing adhered to the ARRIVE guidelines, and before commencement, ethical approval for this study was obtained from the relevant ethics committee (No. 2022KQYX023). The study protocol was reviewed to ensure compliance with ethical standards, and any issues raised were addressed before continuing with data collection.

### 2.1 Preparation of DMAHDM

DMAHDM was synthesized using the Menshutkin method reaction [16]. 10 mmol of 2-(Dimethylamino)ethyl methacrylate (DMAEMA, Macklin, shanghai, China), 10 mmol of Bromohexadecane (BHD, Macklin, shanghai, China), and 3 g of anhydrous ethanol were placed in a brown wide-mouth glass reagent bottle. The bottle was secured with a cap and sealed with a sealing film to ensure an airtight environment. The reagent bottle was then placed in a thermostatic magnetic stirrer heated at 70°C in an oil bath with stirring at 800 rpm for 24 hours after the evaporation of anhydrous ethanol. The resulting DMAHDM is a colorless, transparent, viscous liquid stored away from light [24].

### 2.2 Preparation of DMAHDM-PCL composite fiber

The spinning solution was prepared by adding 1.2 g of Polycaprolactone (PCL, Sigma Aldrich, USA) to 10 mL of Hexafluoroisopropanol (HFIP, Solarbio, Beijing, China) with a mass concentration of 12%. Then, 0 mg, 12 mg, 36 mg, 60 mg, and 84 mg of DMAHDM were added, respectively, and the mixture was magnetically stirred at 1000 rpm for 12 hours to obtain five groups of mixed solutions with mass fractions of 0%, 1%, 3%, 5%, and 7%. These five solution groups were drawn into a disposable syringe, which was connected to a needle with a blunt tip (model 22G). The needle was connected to a high-voltage source, and aluminum foil paper was used as the receiver. The spinning distance was set to 15 cm, the voltage and flow rate were set to 13 kV and 1 mL/h, respectively. The spinning solution was ejected to form a fibrous film and deposited on the receiver under the influence of the electric field [21].

### 2.3 Fourier transform near-infrared (FT-IR) spectroscopic analysis

In order to clarify whether DMAHDM is synthesized and loaded, Fourier transform near-infrared spectroscopy was used to perform infrared analysis on the composite DMAHDM-PCL fibers. After processing with Origin2019 software, observe the absorption peak of the reaction product in the infrared spectrum within the range of 500-4000cm^-1^.

### 2.4 Scanning Electron Microscope (SEM) observation of morphology

The fiber film was cut into 5×8 mm specimens, affixed to a copper sheet using a conductive adhesive, and processed using a fully automated ion-sputtering gold sprayer (20 mA, 30 s). The surface morphology of the fibers was observed and captured using a Gemini SEM 300 instrument (Carl Zeiss Jena, Germany) operating at 10 kV. Fiber diameters were measured using Image-J image analysis software. Forty fibers were sampled from each specimen, and the average diameter and standard deviation were calculated.

### 2.5 Composite fiber membrane antibacterial performance testing

The antimicrobial properties of the composite fibers were evaluated using an agar diffusion test. The fibers were cut into circular specimens of 20 mm diameter and sterilized under UV light for 2 hours. Take 10 μL of *Streptococcus mutans* (ATCC 25175) solution (colony count of 10^7^ CFU/mL) and spread it evenly on the agar medium. Place the sterilized specimen in the center of the agar plate and incubate for 24 to 48 hours at 37°C in a constant temperature incubator. Following incubation, the size of the inhibition zone was observed and measured. The diameter of the antibacterial area was determined using Image-J image analysis software. Each sample was measured 10 times in different directions to minimize error, and the antibacterial zone’s average diameter and standard deviation were calculated.

### 2.6 Preparation of orthodontic adhesive

Fold the fiber membrane into 1–2 cm, freeze embedding, fixation, along the direction perpendicular to the fiber cut into a certain thickness of thin slices. Disperse with distilled water and centrifuge the fibers in a centrifuge at 1000 rpm for 5 minutes and repeat 3 times. The upper layer of the solution was removed and dried to obtain short fiber powder.

Short fibers were added to the orthodontic adhesive Transbond XT (3M, USA) in different mass fractions (0, 1%, 3%, and 5%). The staple fibers and 3M Transbond XT were mixed homogeneously for 3 minutes in a dark room, and the light was cured to form a round specimen 1 cm in diameter and 0.5 mm thick. The edges of the specimen were polished and smooth. The samples were magnetically stirred in distilled water at 100 rpm for 1 h to remove uncured monomers [25]. The specimens were soaked in 75% alcohol for 3 minutes, placed in a 24-well plate, and irradiated with UV light on both sides for 30 minutes before use.

### 2.7 Evaluation of antimicrobial activity by Colony-forming unit (CFU)

The experimental and control groups’ specimens (n = 3) were placed in 24-well plates, and 1 mL of *Streptococcus mutans* solution with a concentration of 1×10^7^ CFU/mL was added. The 24-well plates were placed in a thermostatic incubator and incubated at 37°C with 5% CO_2_ for 6 hours. The two groups of specimens were removed separately with sterile forceps. The resin flakes were gently rinsed three times with Phosphate buffer saline (PBS) and transferred to a centrifuge tube containing 1 mL of PBS. The tube was sonicated for 3 minutes to disperse the biofilm into the suspended bacteria. Dilute the co-cultured bacterial solution and the sonicated bacterial suspension sequentially, and pipette 10 μL of each dilution onto a Brain Heart Infusion Broth (BHI) agar plate, titrating each concentration three times. After the medium was dry and no longer flowed, the plate was inverted and incubated at 37°C with 5% CO2 for 24 h. The bacterial growth was observed, and CFU colonies were counted. After 24 hours, observe the bacterial growth and count the CFU colonies.

### 2.8 Bacterial biofilm activity by Methylthiazolyldiphenyl-tetrazolium bromide (MTT) method

To simulate the aging environment in the oral cavity, the specimens were soaked using artificial saliva, which was replaced every 3 days. After 1 month of soaking, four sets of resin flakes (n = 5) were irradiated with UV light on both sides for 30 minutes. The specimens were transferred to a 24-well plate and incubated with 1 mL of 1×10^7^ CFU/mL *Streptococcus mutans* suspension for 24 hours at 37°C and 5% CO_2_ to form a *Streptococcus mutans* biofilm. The resin flakes were rinsed three times with PBS buffer to remove unattached bacteria and transferred to a new 24-well plate. The bacterial metabolic activity of the biofilm on the resin flakes was analyzed using an MTT kit (Solarbio) [26]. After inoculation with 1 mL of MTT solution (0.5 mg/mL MTT) incubated at 37°C, 5% CO_2_ for 2 hours, 1 mL of dimethyl sulfoxide (DMSO) was added. After incubation at 37°C, 5% CO_2_ for 20 minutes, 100 μL of DMSO solution was transferred to a 96-well plate, and an enzyme marker measured the 540 Optical density (OD_540_) value. The relative biofilm viability (RBV) was calculated using the following formula:

RBV=absorbanceofexperimentalgroup/controlgroupabsorbancex100%

### 2.9 The biocompatibility of the new orthodontic adhesive was evaluated by the Acridine Orange/Ethidium Bromide (AO/EB) method

8×10^4^ mouse fibroblasts (L-929) were inoculated in each well of a 24-well plate and cultured at 37°C and 5% CO_2_ for 24 hours. The experimental groups were supplemented with 200 μL of extracts treated with resin test pieces in five groups of three replicate wells. The experimental group added 200 μL of resin test piece-treated extract with 3 replicate wells per group. 24 hours later, the cells were washed twice with PBS buffer, and 100 μL of PBS buffer was added. The cells were stained with AO/EB (Solarbio, Beijing, China) staining solution, the fluorescent staining solution was discarded, 100 μL PBS buffer was added, and the number of live and dead cells was observed under the fluorescence microscope.

### 2.10 Cell Counting Kit-8 (CCK-8) method to detect the biosafety of orthodontic adhesive

The experimental resin flakes of each group were incubated in Dulbecco’s modified eagle medium (DMEM) containing 10% Fetal bovine serum (FBS) for 24h at room temperature to obtain the extracts. L929 cells were inoculated in 96-well plates at 8×10^3^ cells per well and incubated at 37°C, 5% CO_2_ for 24 h. After removing the medium and washing with PBS 3 times, the treated extracts from the experimental specimens were dropped into 96-well plates separately (100 μL per well, n = 5 per extract), and the cells with pure medium alone were used as a blank control group for natural culture. The 96-well plates were incubated at 37°C and 5% CO_2_ for 24 hours. Drop 10 μL of CCK-8 solution and 90 μL of culture medium mixture into each well. After 2 hours, the absorbance value of each well at 450 nm was measured with an enzyme marker to assess cell viability.


Relativecellviability(%)=(ODofexperimentalgroup‐ODofblank)/(ODofblankcontrolgroup‐ODofblank)x100%

### 2.11 Orthodontic adhesive bond strength and residual index analysis

Forty isolated first premolar teeth extracted due to orthodontic treatment were randomly divided into 4 groups (n = 10) for bond strength testing [27], which had been approved by the local ethical committee under the protocol (No. 2022KQYX023). The enamel surface was cleaned with a brush, and the isolated tooth was embedded in a 10 mm × 8 mm × 8 mm mold with self-coagulating resin and the crown exposed. The isolates were stored in deionized water in a refrigerator at 4°C, and the deionized water was changed weekly. The central buccal surfaces of the crowns were etched by acid etching with 35% phosphoric acid for 30 s. The brackets were bonded with each group’s materials, and the position was adjusted so that the bottom of the upper edge of the brackets was in line with the ground plane Parallel. After changing the position, a dynamometer was used to assist in bonding the brackets in place. After removing more than the bonding agent, a light-curing lamp was used to light-cure the brackets at 1 mm from the enamel surface in the bonding area for 20 s from near to center, for a total of 40 s, with an intensity of 1100 mW/cm^2^. The prepared samples were placed in an artificial saliva immersion treatment for 1 month to simulate aging conditions. The teeth were fixed on the universal testing machine, and the loading speed was set at 0.5 mm/min. The loading head was adjusted so the brackets were directly aligned laterally, and loading was started until the brackets were dislodged. The experiment’s maximum loading (N) was recorded, and the shear bond strength (SBS) was calculated for each group. The bottom area of the orthodontic bracket mesh was approximately 12.97 mm^2^, and the bond strength was calculated according to the following formula:

Bonding strength (MPa) = maximum load (N)/bottom area of bracket mesh (mm^2^)

After the brackets were dislodged, the enamel surface was observed using a stereomicroscope, and the adhesive remnant index (ARI) was evaluated and recorded according to the scoring criteria [28].

Adhesive resident index scoring criteria:

0 = no adhesive residue on the tooth.

1 = less than 50% adhesive residue on the teeth

2 = more than 50% adhesive residue on the tooth.

3 = All adhesive residue is on the enamel surface.

### 2.12 Animal oral mucous membrane irritation test

Animal experiments for biological evaluation according to ISO 10993–10:2009 were approved by the local Human Research Ethics Committee (No. 2022KQYX023). Composite nanofiber (3 wt%) Transbond adhesive was added to the experimental group, and Transbond adhesive was added to the control group. Circular specimens with a diameter of 5 mm and a thickness of 0.5 mm were made, and the surfaces were polished to make the edges smooth and rounded. Holes were punched in the center of the discs to allow sutures to pass through and sterilized for spare use. 15 healthy adult male LVG Hamsters, aged 60 years and weighing 120g-140g, were used for the oral mucosal irritation test. The mice were anesthetized by intraperitoneal injection of 2% pentobarbital (40 mg/Kg). Minimally invasive methods were chosen to minimize pain and suffering for the animals. After disinfection with iodophor for 5 minutes, the test and control materials were sutured into the golden gopher’s buccal mucosa of the oral cavity. The suture force was appropriate to close the specimen to the mucosa without compression. The left side was the control material, the right was the test material, and all the mice were fed regularly with water and diet. The health and behavioral performance of the animals were also closely monitored during the experiment. The mice were executed after 14 days of feeding, and gross changes at the contact site were observed when the material was removed. In order to alleviate the pain of the mice, mice were executed using anaesthesia followed by neck dislocation. The local tissues, including the surrounding tissues, were fixed in a 4% paraformaldehyde solution, sectioned, and stained with hematoxylin and eosin for cytological and histological examination. Biological evaluation according to the requirements of ISO 10993–10:2009 and ISO 7405 Technical Report, grading the degree of response of the oral mucosal tissues according to the irritation index. As follows:

Stimulus index 0–4: no reaction.

Stimulus index 5 to 8: Mild reaction.

Stimulus indexes 9 to 11: moderate reaction.

Stimulus indices 12 to 16: severe reaction.

### 2.13 Statistical analysis

Statistical analyses and graphs were performed using GraphPad Prism 8 statistical software. All values were averaged at least three times and expressed as mean ± standard deviation. One-way analysis of variance (ANOVA) and least significant difference method t-test (LSD-t) were used to compare the differences between groups, and *P* < 0.05 was considered statistically significant.

## 3 Results and discussion

Enamel demineralization is a common complication in fixed orthodontic treatment, primarily induced by changes in the oral microenvironment resulting from orthodontic interventions [29]. Fixed orthodontic appliance grooves and bonding agents can alter the surface roughness of neighboring teeth, increasing the number of adhesion sites that facilitate plaque formation [30]. Standard measures to prevent enamel demineralization include tooth brushing, mouthwash [31], and the use of fluoride and its preparations [32, 33]. In this study, we developed an antimicrobial orthodontic adhesive by preparing DMAHDM and PCL into micro/nanostructured composite fibers by electrostatic spinning technology and adding them to a commercial adhesive. We investigated their effects on bracket bond strength and biofilm resistance. It was shown that adding DMAHDM to orthodontic adhesives did not affect the enamel bond strength. The addition of DMAHDM to orthodontic adhesives produced effective anti-biofilm activity, and the antimicrobial properties of the adhesives were enhanced with the increase of DMAHDM content.

### 3.1 Fourier Infrared (FT-IR) spectral analysis

**Fig 1:**
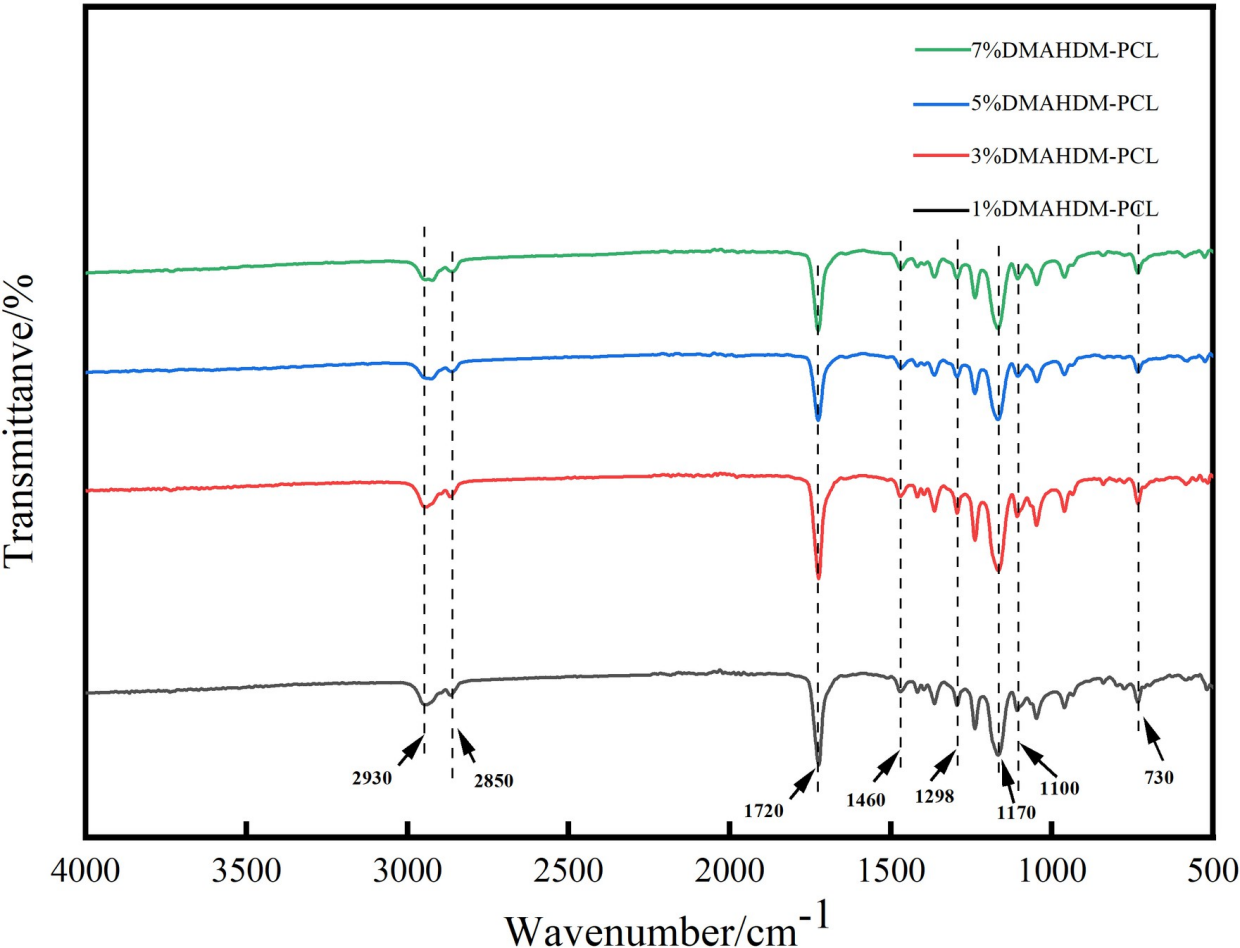
Infrared spectral analysis of DMAHDM-PCL fibers.

The present study synthesized the quaternary monomer DMAHDM using the Menschutkin method [16] with a carbon chain length (CL) of 16. FT-IR can detect the functional groups of unknowns and determine the chemical structure. According to the study [16], the IR spectra of compounds containing NR_4_^+^ groups have peak bands in the region from 1100 cm^-1^ to 450 cm^-1^. In Fig 1, the characteristic peaks of the quaternary ammonium group are 1100 cm^-1^ to 450 cm^-1^, 2930 cm^-1^ and 2850 cm^-1^ are—CH_2_ symmetric and antisymmetric vibration peaks, 1720 cm^-1^ is—C = O—vibration peak, 1460 cm^-1^ is—CH_2_- and—CH_3_ vibration peak, 1298 cm^-1^ is the characteristic peak of C-N in the quaternary ammonium group, 1170 cm^-1^ is the ester C-N vibration peak, and 730 cm^-1^ is the absorption peak of the long chain—CH2 plane rocking vibration. These peaks correspond to the chemical formula of DMAHDM. In addition, no C-Br absorption peak was found in BHD, and no—N (CH_3_) _2_ band was observed in DMAEMA, indicating that the bromine group in BHD successfully reacted with the amine group in DMAEMA to form a quaternary ammonium group. The results indicate that DMAHDM has been successfully synthesized and loaded via electrospinning without damaging its chemical structure. It is attached to the DMAHDM-PCL nanofiber scaffold through physical bonding. DMAHDM bound in this manner is more easily released from the fibers to kill bacteria.

### 3.2 Characterization of composite fiber membranes

**Fig 2:**
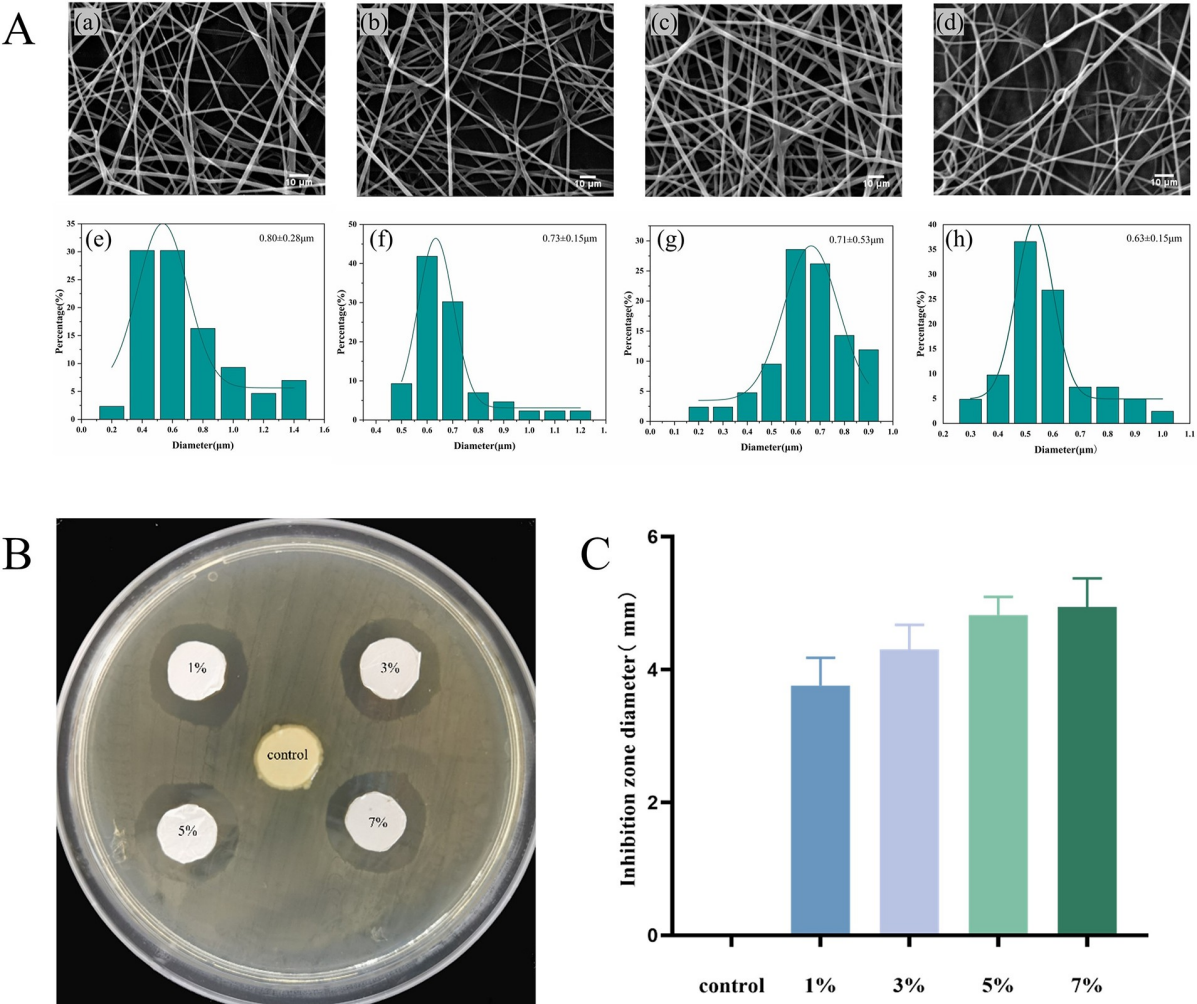
**A.** (a-d) shows the SEM photographs of the electrostatically spun DMAHDM-PCL fibers at DMAHDM mass fractions of 1%, 3%, 5%, and 7%, while (e-h) shows their corresponding fiber diameter distributions and frequency distributions. **B.** It displays pictures of antibacterial rings of specimens with different concentrations of DMAHDM-PCL fibers. **C.** It illustrates the antimicrobial effect of composite fibers with different DMAHDM contents against *Streptococcus mutans* as determined by the agar petri dish diffusion method.

Fig 2A(a-d) shows the SEM images of the electrostatically spun DMAHDM-PCL fibers. The average diameter of the fibers exhibited a decreasing trend with the addition of 1%, 3%, 5%, and 7% DMAHDM, with average diameters of 0.80 μm, 0.73 μm, 0.71 μm, and 0.63 μm, respectively. The surface of the fibers in each group appeared smooth, with no obvious beading or adhesion. Scanning electron microscopy analysis demonstrated favorable fiber morphology.

Fig 2B displays photos of the antibacterial rings formed after 24 hours of co-culture with *Streptococcus mutans* at different DMAHDM concentrations, in the case of 0% composite fibers, no antibacterial ring formation was observed, and bacteria accumulated on the surface of the fibers. The diameter of the antibacterial ring of the fibers tended to increase with the increase in DMAHDM content. Fig 2C illustrates the differences in the size of the antibacterial rings: 0% DMAHDM/PCL < 1% DMAHDM/PCL < 3% DMAHDM/PCL < 5% DMAHDM/PCL < 7% DMAHDM/PCL. Group differences were statistically significant (*p* < 0.05) except for groups d and e.

As the DMAHDM concentration increased, a clear trend of decreasing fiber diameter was observed. The reason for this analysis may be attributed to the fact that the addition of DMAHDM reduces the viscosity of the solution, allowing the spinning solution to stretch the fibers finer in the presence of an electric field. The antimicrobial properties of DMAHDM-PCL fibers were confirmed through composite fiber membrane antibacterial performance testing. It was found that there was no significant difference in the size of the inhibitory ring presented by fibers with 5% and 7% additions. In order to avoid increased toxicity and cost due to excessive addition of DMAHDM, this study will utilize the maximum antimicrobial properties achieved with 5% DMAHDM-PCL composite fibers for subsequent exploration.

### 3.3 Antibacterial properties of the orthodontic adhesive

**Fig 3:**
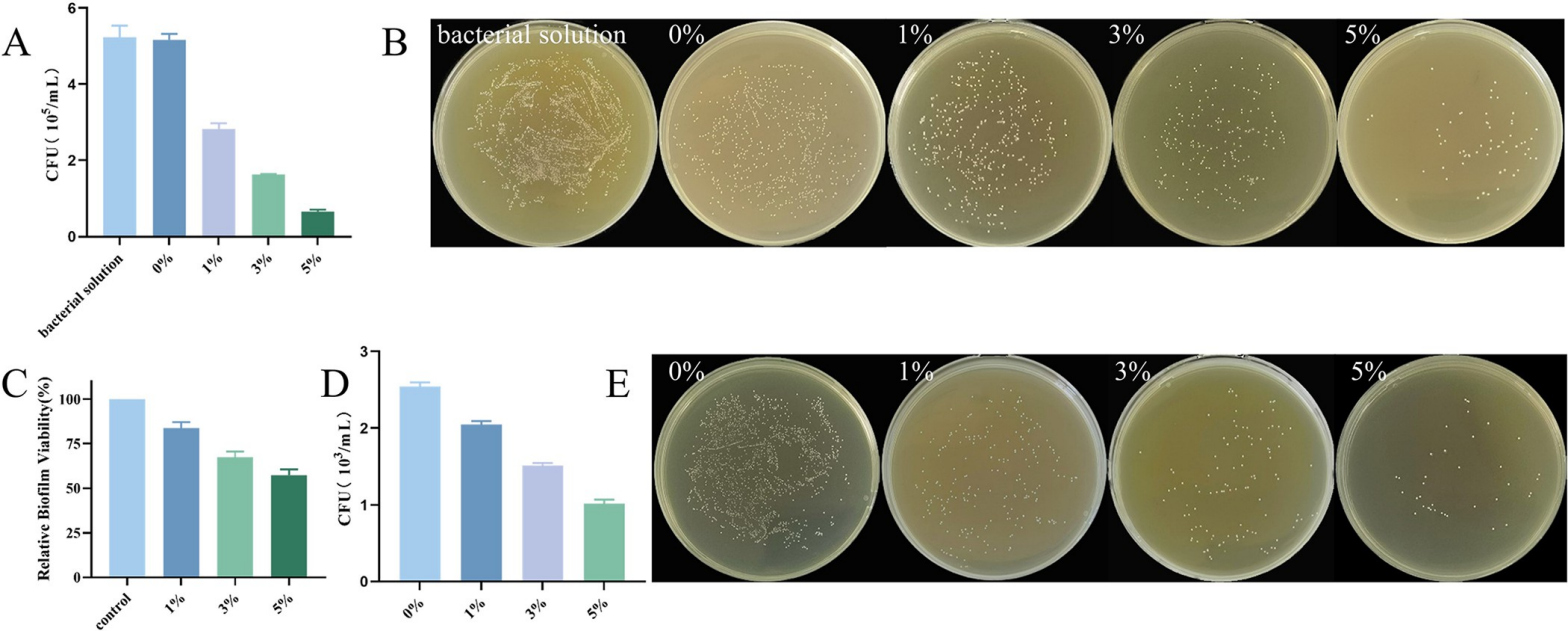
**A.** Bacterial CFU counts in the leachate in the resin flakes after co-culturing *Streptococcus mutans* and the resin flakes for 6 hours. **B.** Pictures of bacterial colonies in the bacterial solution, 0%, 1%, 3%, and 5% extracts after dilution of 10^5^. **C.** Graph of results of relative biofilm activity determined by MTT method. **D.** CFU counts of bacterial biofilms adhering to the surface of resin flakes after co-culture of *Streptococcus mutans* and resin flakes for 6 hours. **E.** Graph of bacterial biofilm CFU counts on the surface of the resin flakes after co-culturing 3M Transbond XT adhesive, 1%, 3%, and 5% resin flakes for 6 hours after diluting 10^3^.

Fig 3A: The results show no significant difference between the bacterial growth in the extract and bacterial suspension of the control group (*P* > 0.05). However, the bacterial growth in the resin flakes extract of the experimental group was lower than that of the control group and bacterial suspension, with results showing a significant difference (*P* < 0.05). Furthermore, the bacterial growth in the experimental group (1%, 3%, 5%) gradually decreased, with statistically significant differences between all groups (*P* < 0.05). Fig 3B: The pictures display biofilm CFU counts of resin flakes extracts and bacterial suspensions for drop plate, 3M Transbond XT adhesive, 1%, 3%, 5% DMAHDM-PCL resin flakes cultured on the resin flakes for 6 hours after dilution of 10^5^. The number of bacterial colonies decreased to varying degrees with increased antimicrobial fibers.

Fig 3C: MTT results of relative biofilm viability show that the antimicrobial activity of the orthodontic adhesives in the experimental group was significantly higher than that of the control group (0% group). Compared with the control group, there was a statistically significant decrease in the metabolic activity of *Streptococcus mutans* biofilm in the experimental group (*P* < 0.05). Moreover, the metabolic activity of *Streptococcus mutans* biofilm showed a decreasing trend with the increase in the amount of antimicrobial material added, and the difference between them was statistically significant (*P* < 0.05).

Fig 3D: The results reveal that the bacterial growth of the resin flakes in the experimental group was lower than that of the control group, with a significant difference (*P* < 0.05). The bacterial growth of the experimental group (1%, 3%, 5%) gradually decreased, with statistically significant differences between all groups (*P* < 0.05). Fig 3E: Bacteria adhering to the surface of the resin flakes were subjected to a drop plate, and the pictures display biofilm CFU counts on 3M Transbond XT adhesive, 1%, 3%, 5% DMAHDM-PCL resin flakes cultured on the surface of the resin flakes for 6 hours after dilution of 10^3^. The number of colonies decreased to varying degrees with increased antimicrobial fibers.

*Streptococcus mutans*, known for its acidophilic and acidic nature, is considered one of the main causative agents of enamel demineralization [34]. In this experiment, the bacterial biofilm study by CFU counting assay for the bacterial biofilm adhering to the surface of the material showed that the bacterial biofilm metabolism level of the resin sheet containing 1%, 3% and 5% fibers was reduced by 20%, 41% and 60%, respectively, as compared to the control group. Due to the advantages of high porosity and large specific surface area of the nanofibers, with the increase in the content of drug-carrying nanofibers, the bacterial-drug contact area was increased, resulting in an enhanced surface antimicrobial effect. For the study of bacterial concentration in the extract, the level of bacterial biofilm metabolism was reduced by 45%, 68%, and 87% for resin sheets containing 1%, 3%, and 5% fibers, respectively, as compared to the Transbond XT control. Over time, a portion of the DMAHDM can be released into the surrounding environment, providing a long-distance antimicrobial effect on areas around the bracket where bacteria tend to attach. When negatively charged bacterial cells are exposed to positively charged quaternary N+ ions, the charge balance is disturbed and the bacteria can rupture under their own osmotic pressure [35]. The MTT method, which assesses bacterial inhibition after 1 month of aging treatments, reveals a similar trend, with a more pronounced antimicrobial effect as the concentration of DMAHDM-PCL fibers increases. Previous studies have shown that some of the released DMAHDM can copolymerize with the resin, becoming immobilized in the material and not leaching out over time. This provides long-lasting antimicrobial and mechanical properties [36, 37]. It was shown that after 6 months of water aging, the antimicrobial activity of quaternary ammonium salt antibacterial monomers did not change significantly [38]. However, the oral environment is a complex ecosystem with diverse flora, so it is necessary to investigate the antimicrobial properties of novel adhesives in subsequent experiments that simulate multiple types of bacteria in the intraoral environment.

### 3.4 Biosafety performance of the orthodontic adhesive

**Fig 4:**
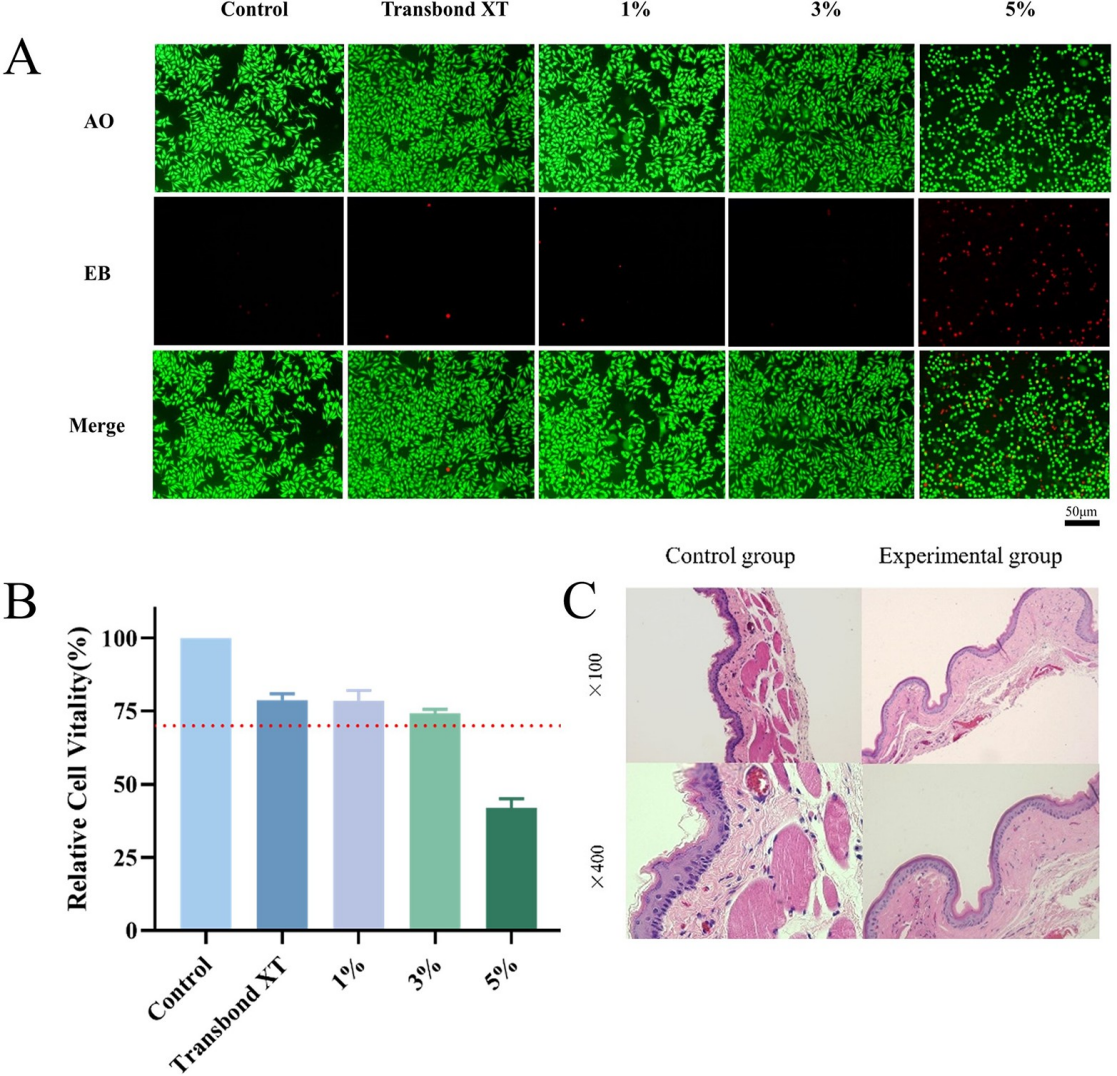
**A.** Fluorescence microscopy images of the orthodontic adhesive co-cultured with L-929 for 24 hours after AO/EB staining. **B.** Relative viability of L929 cells between groups detected by the CCK8 method. **C.** HE staining of oral mucosa tissues (100×, 400×).

Fig 4A displays images analyzed by acridine orange/ethidium bromide (AO/EB) double staining after co-culturing with L-929 cells for 24 hours under a fluorescence microscope. After cell inoculation, the co-culturing was performed at various mass fractions of composite fiber addition, including 0%, 1%, 3%, and 5%. Acridine orange (AO) can enter the nuclei of living cells, emitting green fluorescence, while ethidium bromide (EB) can only enter the nuclei of apoptotic cells, emitting red fluorescence [39]. The images reveal a large number of green cells with normal morphology and structure after AO staining in all groups except the 5% group and a smaller number of red apoptotic cells under EB staining. In contrast, the 5% group exhibited a large number of cells with greenish nuclear chromatin in the form of condensation or round balls after AO staining, and the number of red apoptotic cells was also higher compared to the other groups. Fig 4B: CCK8 relative cell viability assay showed no significant difference in relative cell viability between the 1% and 3% groups and the Transbond XT group (*P* < 0.05), and the relative cell viability of all three groups was higher than 70%. However, there was a statistically significant difference between the 5% group and all groups, and the relative cell viability was lower than 50%.

Regarding the postoperative period at 14 days, 11 out of 15 mice in both groups did not experience material dislodgement. The mucosal surface of the buccal sac in direct contact with the test and control materials appeared pink in color, with no obvious erosion, ulceration, congestion, or edema. Fig 4C shows the pathological observation of the buccal bursa mucosa. The tissue section of the buccal bursa mucosa in the experimental group exhibited complete and continuous epithelial tissue, clear cellular layers, and structure, with no abnormal changes such as nail protrusion and overgrowth. No significant inflammatory cell infiltration within the connective tissue and no obvious submucosal congestion were observed. Table 1 Grading of Pathological Reactions (ISO 10993-1-2009) indicates that the experimental materials do not cause oral mucosal irritation.

**Table 1:** Grading of pathological reactions.

**Serial Number**	1	2	3	5	7	8	10	11	13	14	15
**Reaction Score**	2	3	4	2	3	1	0	2	3	4	5
**Hierarchy**	No	No	No	No	No	No	No	No	No	No	Mild reaction

Quaternary ammonium salts are widely used in oral materials [15, 17, 18] due to their low toxicity properties [40]. In most studies [15, 17, 18, 25, 41], the resin composites retained their mechanical properties without examining toxicity. Therefore, as a biomedical material, it is crucial to evaluate the biocompatibility of DMAHDM-PCL-containing materials before clinical translation. The amount of DMAHDM-PCL fibers added within the biosafety range in this study needs clarification. We used L929 mouse fibroblasts and obtained test material extracts for cytocompatibility testing as a culture medium. A cell viability of 70% is the criterion for non-cytotoxicity [42]. CCK-8 results demonstrated that cell viability was higher than 70% in all groups except for the 5% group. The 1% and 3% nanofibers in the DMAHDM-PCL group were not statistically different from the control group. Therefore, the 1% and 3% content of DMAHDM-PCL fibers were more in line with the criteria and had good cytocompatibility and high antimicrobial properties. AO/EB staining further confirmed the biosafety of the 3% addition, whereas more than 5% of the material affected the normal morphology and number of cells. We, therefore, finalized the biosafety range of DMAHDM-PCL with a 3% addition. To further investigate the material’s biocompatibility, we tested the oral mucosal irritation of golden gophers within 14 days. The results showed that the irritation response of the test group to the mucosa of the buccal vesicle of the mice in the short term was the same as that of the control group. No noticeable epithelial changes, inflammatory reactions, or inflammatory cell infiltration were observed, indicating that the composite fiber resin-containing adhesive is non-irritating to the oral mucosa and has good biosafety. Evaluating the biocompatibility of a new material should include several tests, including primary and secondary tests. In this study, only a preliminary evaluation of short-term mucosal irritation was conducted, and exact conclusions require more biological tests in conjunction with human application.

### 3.5 Mechanical properties of new orthodontic adhesive

**Fig 5:**
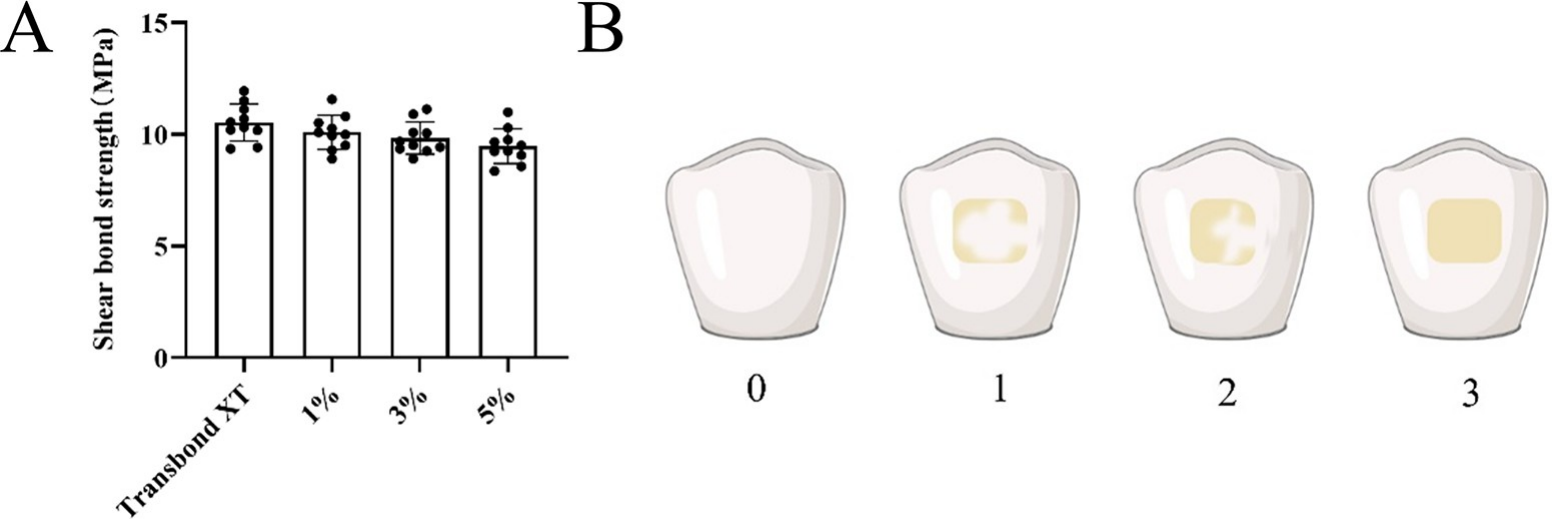
**A.** SBS values for each group of adhesives. **B.** Schematic diagram of the categories of adhesive residual index.

As shown in Fig 5A, there was no statistically significant difference in bond strength between the groups (*P* > 0.05), except for a statistically significant difference (*P* < 0.05) between the Transbond XT and Transbond XT+5% DMAHDM-PCL groups. Table 2 shows that the bond strength of the adhesive in the Transbond XT group was 10.53±0.83 MPa. The adhesive bond strength of the experimental group was 10.09±0.77 MPa, 9.83±0.72 MPa, and 9.47±0.77 MPa, respectively. The minimum values of adhesive bond strength for each group were 9.36 MPa, 8.91 MPa, 8.91 MPa, and 8.36 MPa, all higher than 8 MPa. The ARI scores in Table 3 showed that the scores of each group were evenly distributed, and their scores were mainly distributed between 1 and 2, indicating that bond damage mainly occurs between adhesives. There is no significant difference between the groups (*P* > 0.05).

**Table 2:** SBS values (n = 10) of orthodontic adhesive for each group.

Groups	Number	SBS (Mpa)		
		Mean±SD	Min	Max
**Transbond XT**	10	10.53±0.83	9.36	11.95
**TransbondXT+1%DMAHDM-PCL**	10	10.09±0.77	8.91	11.57
**TransbondXT+3%DMAHDM-PCL**	10	9.83±0.72	8.91	11.13
**TransbondXT+5%DMAHDM-PCL**	10	9.47±0.77	8.36	10.99

**Table 3:** Residual index (ARI) scores of orthodontic adhesive for each group (n = 10).

Groups	ARI scores				Total score
	0	1	2	3	
**Transbond XT**	2	3	4	1	14^a^
**Transbond XT+1%DMAHDM-PCL**	0	4	5	1	17^a^
**Transbond XT+3% DMAHDM-PCL**	1	3	4	2	17^a^
**Transbond XT+5% DMAHDM-PCL**	1	4	3	2	16^a^

Note: Groups with the same letter are not statistically significantly different (*P* > 0.05).

Using a new bonding agent should achieve a certain level of bond strength. If the bond strength is excessively high, it may damage enamel during bracket removal. Conversely, if the bond strength is too low, it will fail to meet clinical requirements, potentially resulting in bracket dislodgement [21, 43]. Tuncer suggested that the minimum bond strength of orthodontic adhesive in clinical practice should be 8–9 MPa [44]. Under the experimental conditions of this study, which involved the addition of nanofibers as fillers to commercial bonding agents, the mean bond strength values of the experimental groups exhibited a slight decreasing trend. The experimental groups achieved similar bond strengths in orthodontic adhesive as the control group, with shear bond strengths ranging from 8.36 to 11.57 MPa. The SBS values of all the groups exceeded 8 MPa, which is acceptable and meets the clinical criteria for bonding.

The ARI index is an auxiliary measure for assessing the bonding effect between the adhesive and the enamel surface. It provides an indirect evaluation of adhesive bond strength and the location of the fracture surface [45]. A higher ARI index indicates that the fracture surface is closer to the bracket adhesive interface, making bracket removal safer. Pont et al. [46] concluded that a lower ARI index suggests that the fracture predominantly occurs in the area between the enamel and the bonding agent, which may potentially cause damage to the tooth during bracket dislodgment. In this study, the ARI scores of the experimental and control groups were concentrated at 1–2. This indicates that the fracture surfaces of bracket dislodgment were similar between the two groups, both characterized by fractures within the bonding agent. The adhesive bond between the bonding agent and the tooth surface was safer while achieving the required clinical adhesive strength for the brackets. This effectively reduces the risk of enamel exfoliation during bracket removal.

However, there are some limitations to this study. These include the extended duration of orthodontic treatment, the long-term antimicrobial properties of the new bonding agent, and whether the presence of antimicrobial materials affects its long-term bond strength. Further investigation is required to address these issues. Additionally, Evaluating the biocompatibility of novel materials needs to ensure that the evaluation process is comprehensive to guarantee their safety in clinical applications and that there are differences between humans and animals. The present study only provides a preliminary assessment of the biocompatibility of the novel materials, and a comprehensive evaluation of the novel materials requires further study in conjunction with other biological tests.

## 4. Conclusions

This study attempted to determine the optimal concentration of DMAHDM-PCL nanofiber content in orthodontic adhesives to balance antimicrobial activity, adhesive properties, and biosafety. It was finally concluded that DMAHDM-PCL nanofibers containing 3 wt% of DMAHDM-PCL nanofibers could improve antimicrobial properties, as evidenced by reduced biofilm activity and colony counts. Simultaneously, they met clinical bonding requirements and biosafety criteria. The application of this novel antimicrobial material is expected to help inhibit bacteria during orthodontic procedures and may also inspire antimicrobial applications of other resin-based materials.

## Supporting information

S1 File This is the raw data of Fourier Infrared (FT-IR) spectral analysis. (ZIP)

S2 File This is the raw data of characterization of composite fiber membranes. (RAR)

S3 File This is the raw data of antibacterial properties of the orthodontic adhesive. (ZIP)

S4 File This is the raw data of biosafety performance of the orthodontic adhesive. (ZIP)

S5 File This is the raw data of mechanical properties of new orthodontic adhesive. (ZIP)

## References

[1] Brignardello-Petersen R. No differences important to patients between orthodontic treatment with customized fixed appliances and conventional appliances. Journal of the American Dental Association (1939). 2017;148(12):e194. doi: 10.1016/j.adaj.2017.09.015 29029750

[2] Papageorgiou SN, Koletsi D, Iliadi A, Peltomaki T, Eliades T. Treatment outcome with orthodontic aligners and fixed appliances: a systematic review with meta-analyses. European journal of orthodontics. 2020;42(3):331–43. doi: 10.1093/ejo/cjz094 31758191

[3] Heymann GC, Grauer D. A contemporary review of white spot lesions in orthodontics. Journal of esthetic and restorative dentistry: official publication of the American Academy of Esthetic Dentistry [et al]. 2013;25(2):85–95. doi: 10.1111/jerd.12013 23617380

[4] Perdigão J. Resin infiltration of enamel white spot lesions: An ultramorphological analysis. Journal of esthetic and restorative dentistry: official publication of the American Academy of Esthetic Dentistry [et al]. 2020;32(3):317–24. doi: 10.1111/jerd.12550 31742888

[5] Maxfield BJ, Hamdan AM, Tüfekçi E, Shroff B, Best AM, Lindauer SJ. Development of white spot lesions during orthodontic treatment: perceptions of patients, parents, orthodontists, and general dentists. American journal of orthodontics and dentofacial orthopedics: official publication of the American Association of Orthodontists, its constituent societies, and the American Board of Orthodontics. 2012;141(3):337–44. doi: 10.1016/j.ajodo.2011.08.024 22381494

[6] Lundström F, Krasse B. Streptococcus mutans and lactobacilli frequency in orthodontic patients; the effect of chlorhexidine treatments. European journal of orthodontics. 1987;9(2):109–16. doi: 10.1093/ejo/9.2.109 3472888

[7] Khachatryan G, Markaryan M, Vardanyan I, Manrikyan M, Manrikyan G. Morphological Characteristics and Prevention of Tooth Enamel Demineralization during Orthodontic Treatment with Non-Removable Appliances. International journal of environmental research and public health. 2022;20(1). doi: 10.3390/ijerph20010540 36612862 PMC9819192

[8] Marinelli G, Inchingolo AD, Inchingolo AM, Malcangi G, Limongelli L, Montenegro V, et al. White spot lesions in orthodontics: prevention and treatment. A descriptive review. Journal of biological regulators and homeostatic agents. 2021;35(2 Suppl. 1):227–40. doi: 10.23812/21-2supp1-24 34281321

[9] Khoroushi M, Kachuie M. Prevention and Treatment of White Spot Lesions in Orthodontic Patients. Contemporary clinical dentistry. 2017;8(1):11–9. doi: 10.4103/ccd.ccd_216_17 28566845 PMC5426141

[10] Kwaśniewska D, Chen YL, Wieczorek D. Biological Activity of Quaternary Ammonium Salts and Their Derivatives. Pathogens (Basel, Switzerland). 2020;9(6). doi: 10.3390/pathogens9060459 32531904 PMC7350379

[11] Tischer M, Pradel G, Ohlsen K, Holzgrabe U. Quaternary ammonium salts and their antimicrobial potential: targets or nonspecific interactions? ChemMedChem. 2012;7(1):22–31. doi: 10.1002/cmdc.201100404 22113995

[12] Buffet-Bataillon S, Tattevin P, Bonnaure-Mallet M, Jolivet-Gougeon A. Emergence of resistance to antibacterial agents: the role of quaternary ammonium compounds—a critical review. International journal of antimicrobial agents. 2012;39(5):381–9. doi: 10.1016/j.ijantimicag.2012.01.011 22421329

[13] Vieira DB, Carmona-Ribeiro AM. Cationic lipids and surfactants as antifungal agents: mode of action. The Journal of antimicrobial chemotherapy. 2006;58(4):760–7. doi: 10.1093/jac/dkl312 16885181

[14] Dan W, Gao J, Qi X, Wang J, Dai J. Antibacterial quaternary ammonium agents: Chemical diversity and biological mechanism. European journal of medicinal chemistry. 2022;243:114765. doi: 10.1016/j.ejmech.2022.114765 36116235

[15] Wang X, Zhang N, Wang B, Park SR, Weir MD, Xu HHK, et al. Novel self-etching and antibacterial orthodontic adhesive containing dimethylaminohexadecyl methacrylate to inhibit enamel demineralization. Dental materials journal. 2018;37(4):555–61. doi: 10.4012/dmj.2017-286 29998940

[16] Antonucci JM, Zeiger DN, Tang K, Lin-Gibson S, Fowler BO, Lin NJ. Synthesis and characterization of dimethacrylates containing quaternary ammonium functionalities for dental applications. Dental materials: official publication of the Academy of Dental Materials. 2012;28(2):219–28. doi: 10.1016/j.dental.2011.10.004 22035983 PMC3259208

[17] Zhang N, Weir MD, Chen C, Melo MA, Bai Y, Xu HH. Orthodontic cement with protein-repellent and antibacterial properties and the release of calcium and phosphate ions. Journal of dentistry. 2016;50:51–9. doi: 10.1016/j.jdent.2016.05.001 27157089

[18] Cao L, Xie X, Wang B, Weir MD, Oates TW, Xu HHK, et al. Protein-repellent and antibacterial effects of a novel polymethyl methacrylate resin. Journal of dentistry. 2018;79:39–45. doi: 10.1016/j.jdent.2018.09.007 30248381

[19] Hong J, Yeo M, Yang GH, Kim G. Cell-Electrospinning and Its Application for Tissue Engineering. International journal of molecular sciences. 2019;20(24). doi: 10.3390/ijms20246208 31835356 PMC6940787

[20] Ingavle GC, Leach JK. Advancements in electrospinning of polymeric nanofibrous scaffolds for tissue engineering. Tissue engineering Part B, Reviews. 2014;20(4):277–93. doi: 10.1089/ten.TEB.2013.0276 24004443

[21] Yuan Q, Zhang Q, Xu X, Du Y, Xu J, Song Y, et al. Development and Characterization of Novel Orthodontic Adhesive Containing PCL-Gelatin-AgNPs Fibers. Journal of functional biomaterials. 2022;13(4). doi: 10.3390/jfb13040303 36547563 PMC9783259

[22] Sun H, Mei L, Song C, Cui X, Wang P. The in vivo degradation, absorption and excretion of PCL-based implant. Biomaterials. 2006;27(9):1735–40. doi: 10.1016/j.biomaterials.2005.09.019 16198413

[23] Lu X, Zou H, Liao X, Xiong Y, Hu X, Cao J, et al. Construction of PCL-collagen@PCL@PCL-gelatin three-layer small diameter artificial vascular grafts by electrospinning. Biomedical materials (Bristol, England). 2022;18(1). doi: 10.1088/1748-605X/aca269 36374009

[24] Murata H, Koepsel RR, Matyjaszewski K, Russell AJ. Permanent, non-leaching antibacterial surface—2: how high density cationic surfaces kill bacterial cells. Biomaterials. 2007;28(32):4870–9. doi: 10.1016/j.biomaterials.2007.06.012 17706762

[25] Wang L, Xie X, Li C, Liu H, Zhang K, Zhou Y, et al. Novel bioactive root canal sealer to inhibit endodontic multispecies biofilms with remineralizing calcium phosphate ions. Journal of dentistry. 2017;60:25–35. doi: 10.1016/j.jdent.2017.02.011 28223198

[26] Benov L. Improved Formazan Dissolution for Bacterial MTT Assay. Microbiology spectrum. 2021;9(3):e0163721. doi: 10.1128/spectrum.01637-21 34937171 PMC8694201

[27] Ravuru D, Vivek Reddy G, Bhupathi A, Sunil Kumar KT, Singaraju GS, Mandava P. Evaluation of Antimicrobial Properties and Shear Bond Strength of Conventional Orthodontic Adhesive Modified With Calotropis gigantea Nanoparticles: An In Vitro Study. Cureus. 2023;15(12):e51182. doi: 10.7759/cureus.51182 38283466 PMC10817711

[28] Yu F, Dong Y, Yu HH, Lin PT, Zhang L, Sun X, et al. Antibacterial Activity and Bonding Ability of an Orthodontic Adhesive Containing the Antibacterial Monomer 2-Methacryloxylethyl Hexadecyl Methyl Ammonium Bromide. Scientific reports. 2017;7:41787. doi: 10.1038/srep41787 28169312 PMC5294631

[29] Salmerón-Valdés EN, Lara-Carrillo E, Medina-Solís CE, Robles-Bermeo NL, Scougall-Vilchis RJ, Casanova-Rosado JF, et al. Tooth demineralization and associated factors in patients on fixed orthodontic treatment. Scientific reports. 2016;6:36383. doi: 10.1038/srep36383 27805027 PMC5090428

[30] Gorton J, Featherstone JD. In vivo inhibition of demineralization around orthodontic brackets. American journal of orthodontics and dentofacial orthopedics: official publication of the American Association of Orthodontists, its constituent societies, and the American Board of Orthodontics. 2003;123(1):10–4. doi: 10.1067/mod.2003.47 12532056

[31] Braga AS, Simas LLM, Pires JG, Souza BM, de Melo F, Saldanha LL, et al. Antibiofilm and anti-caries effects of an experimental mouth rinse containing Matricaria chamomilla L. extract under microcosm biofilm on enamel. Journal of dentistry. 2020;99:103415. doi: 10.1016/j.jdent.2020.103415 32592827

[32] Sonesson M, Brechter A, Abdulraheem S, Lindman R, Twetman S. Fluoride varnish for the prevention of white spot lesions during orthodontic treatment with fixed appliances: a randomized controlled trial. European journal of orthodontics. 2020;42(3):326–30. doi: 10.1093/ejo/cjz045 31197364

[33] Lynch RJ, Navada R, Walia R. Low-levels of fluoride in plaque and saliva and their effects on the demineralisation and remineralisation of enamel; role of fluoride toothpastes. International dental journal. 2004;54(5 Suppl 1):304–9. doi: 10.1111/j.1875-595x.2004.tb00003.x 15509081

[34] Takahashi N, Nyvad B. The role of bacteria in the caries process: ecological perspectives. Journal of dental research. 2011;90(3):294–303. doi: 10.1177/0022034510379602 20924061

[35] Beyth N, Yudovin-Farber I, Bahir R, Domb AJ, Weiss EI. Antibacterial activity of dental composites containing quaternary ammonium polyethylenimine nanoparticles against Streptococcus mutans. Biomaterials. 2006;27(21):3995–4002. doi: 10.1016/j.biomaterials.2006.03.003 16564083

[36] Zhang N, Zhang K, Melo MA, Weir MD, Xu DJ, Bai Y, et al. Effects of Long-Term Water-Aging on Novel Anti-Biofilm and Protein-Repellent Dental Composite. International journal of molecular sciences. 2017;18(1). doi: 10.3390/ijms18010186 28106774 PMC5297818

[37] Bhadila G, Filemban H, Wang X, Melo MAS, Arola DD, Tay FR, et al. Bioactive low-shrinkage-stress nanocomposite suppresses S. mutans biofilm and preserves tooth dentin hardness. Acta biomaterialia. 2020;114:146–57. doi: 10.1016/j.actbio.2020.07.057 32771591

[38] Zhang K, Cheng L, Wu EJ, Weir MD, Bai Y, Xu HH. Effect of water-ageing on dentine bond strength and anti-biofilm activity of bonding agent containing new monomer dimethylaminododecyl methacrylate. Journal of dentistry. 2013;41(6):504–13. doi: 10.1016/j.jdent.2013.03.011 23583528 PMC3751171

[39] Liu K, Liu PC, Liu R, Wu X. Dual AO/EB staining to detect apoptosis in osteosarcoma cells compared with flow cytometry. Medical science monitor basic research. 2015;21:15–20. doi: 10.12659/MSMBR.893327 25664686 PMC4332266

[40] Duarte de Oliveira FJ, Ferreira da Silva Filho PS, Fernandes Costa MJ, Rabelo Caldas MRG, Dutra Borges BC, Gadelha de Araújo DF. A comprehensive review of the antibacterial activity of dimethylaminohexadecyl methacrylate (DMAHDM) and its influence on mechanical properties of resin-based dental materials. The Japanese dental science review. 2021;57:60–70. doi: 10.1016/j.jdsr.2021.03.003 33995712 PMC8102164

[41] Tao S, Su Z, Xiang Z, Xu HHK, Weir MD, Fan M, et al. Nano-calcium phosphate and dimethylaminohexadecyl methacrylate adhesive for dentin remineralization in a biofilm-challenged environment. Dent Mater. 2020;36(10):e316–e28. doi: 10.1016/j.dental.2020.08.001 32847685

[42] Zhou W, Zhao H, Li Z, Huang X. Autopolymerizing acrylic repair resin containing low concentration of dimethylaminohexadecyl methacrylate to combat saliva-derived bacteria. Journal of materials science Materials in medicine. 2022;33(6):49. doi: 10.1007/s10856-022-06670-7 35639209 PMC9156454

[43] Eslamian L, Borzabadi-Farahani A, Karimi S, Saadat S, Badiee MR. Evaluation of the Shear Bond Strength and Antibacterial Activity of Orthodontic Adhesive Containing Silver Nanoparticle, an In-Vitro Study. Nanomaterials (Basel, Switzerland). 2020;10(8). doi: 10.3390/nano10081466 32727028 PMC7466539

[44] Tuncer C, Tuncer BB, Ulusoy C. Effect of fluoride-releasing light-cured resin on shear bond strength of orthodontic brackets. American journal of orthodontics and dentofacial orthopedics: official publication of the American Association of Orthodontists, its constituent societies, and the American Board of Orthodontics. 2009;135(1):14.e1–6; discussion -5. doi: 10.1016/j.ajodo.2008.09.016 19121495

[45] Cheng HY, Chen CH, Li CL, Tsai HH, Chou TH, Wang WN. Bond strength of orthodontic light-cured resin-modified glass ionomer cement. European journal of orthodontics. 2011;33(2):180–4. doi: 10.1093/ejo/cjq056 20805142

[46] Pont HB, Özcan M, Bagis B, Ren Y. Loss of surface enamel after bracket debonding: an in-vivo and ex-vivo evaluation. American journal of orthodontics and dentofacial orthopedics: official publication of the American Association of Orthodontists, its constituent societies, and the American Board of Orthodontics. 2010;138(4):387.e1–.e9. doi: 10.1016/j.ajodo.2010.01.028 20889035

