# Supplementary figures and images for: Preparation and performance evaluation of a novel orthodontic adhesive incorporating composite dimethylaminohexadecyl methacrylate—Polycaprolactone fibers
Source: PLoS One. 2024 May 23;19(5):e0304143. doi: 10.1371/journal.pone.0304143 (PMC11115245; doi:10.1371/journal.pone.0304143)

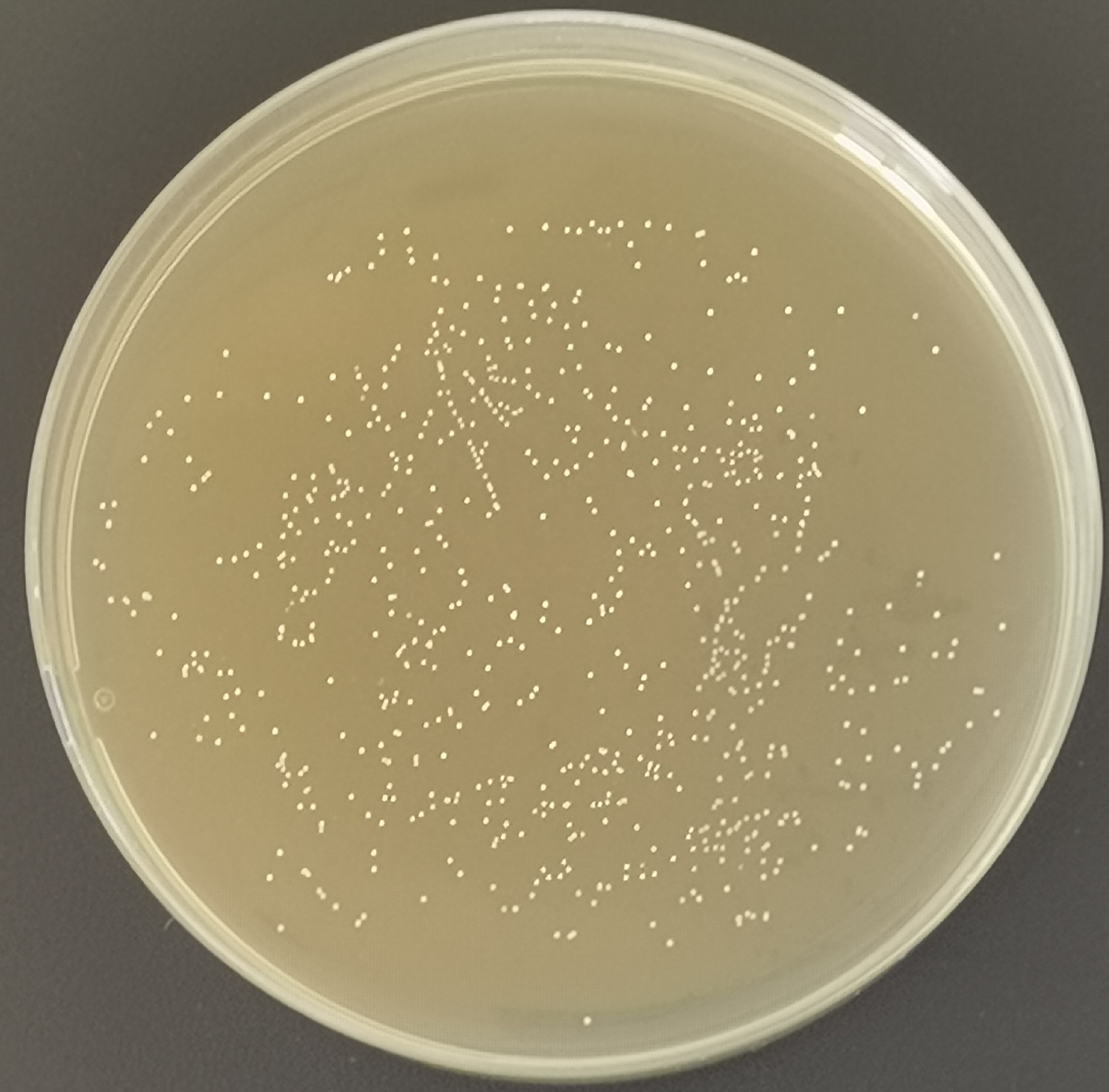

Supplement: S3 File — (ZIP) [file pone.0304143.s003.zip › S3 File.Antibacterial properties of the orthodontic adhesive/CFU/Extracts/0%.jpg]

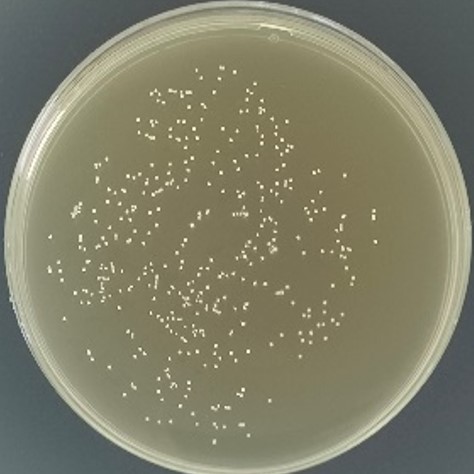

Supplement: S3 File — (ZIP) [file pone.0304143.s003.zip › S3 File.Antibacterial properties of the orthodontic adhesive/CFU/Extracts/1%.jpg]

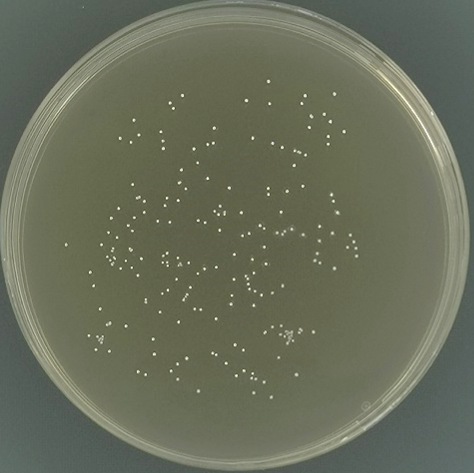

Supplement: S3 File — (ZIP) [file pone.0304143.s003.zip › S3 File.Antibacterial properties of the orthodontic adhesive/CFU/Extracts/3%.png]

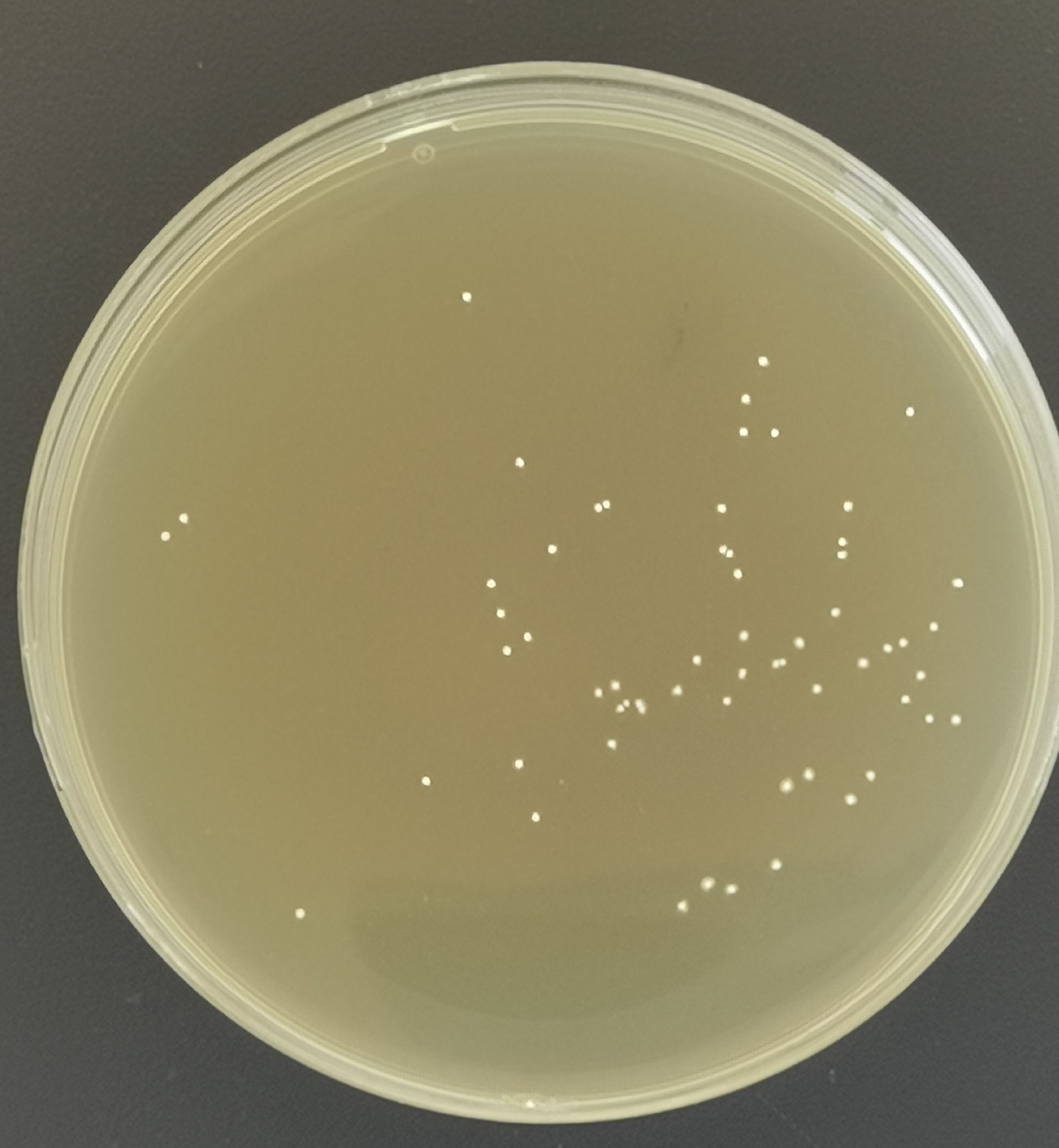

Supplement: S3 File — (ZIP) [file pone.0304143.s003.zip › S3 File.Antibacterial properties of the orthodontic adhesive/CFU/Extracts/5%.jpg]

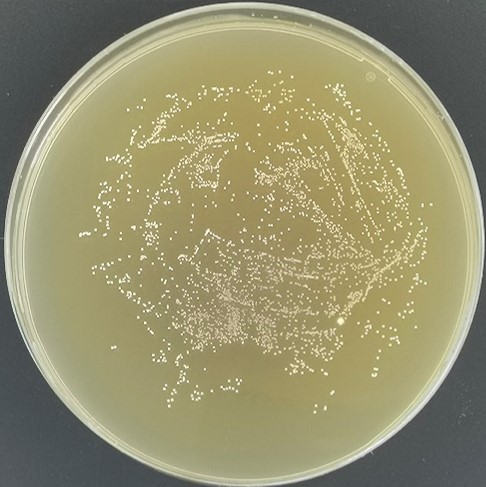

Supplement: S3 File — (ZIP) [file pone.0304143.s003.zip › S3 File.Antibacterial properties of the orthodontic adhesive/CFU/Extracts/bacterial solution.jpg]

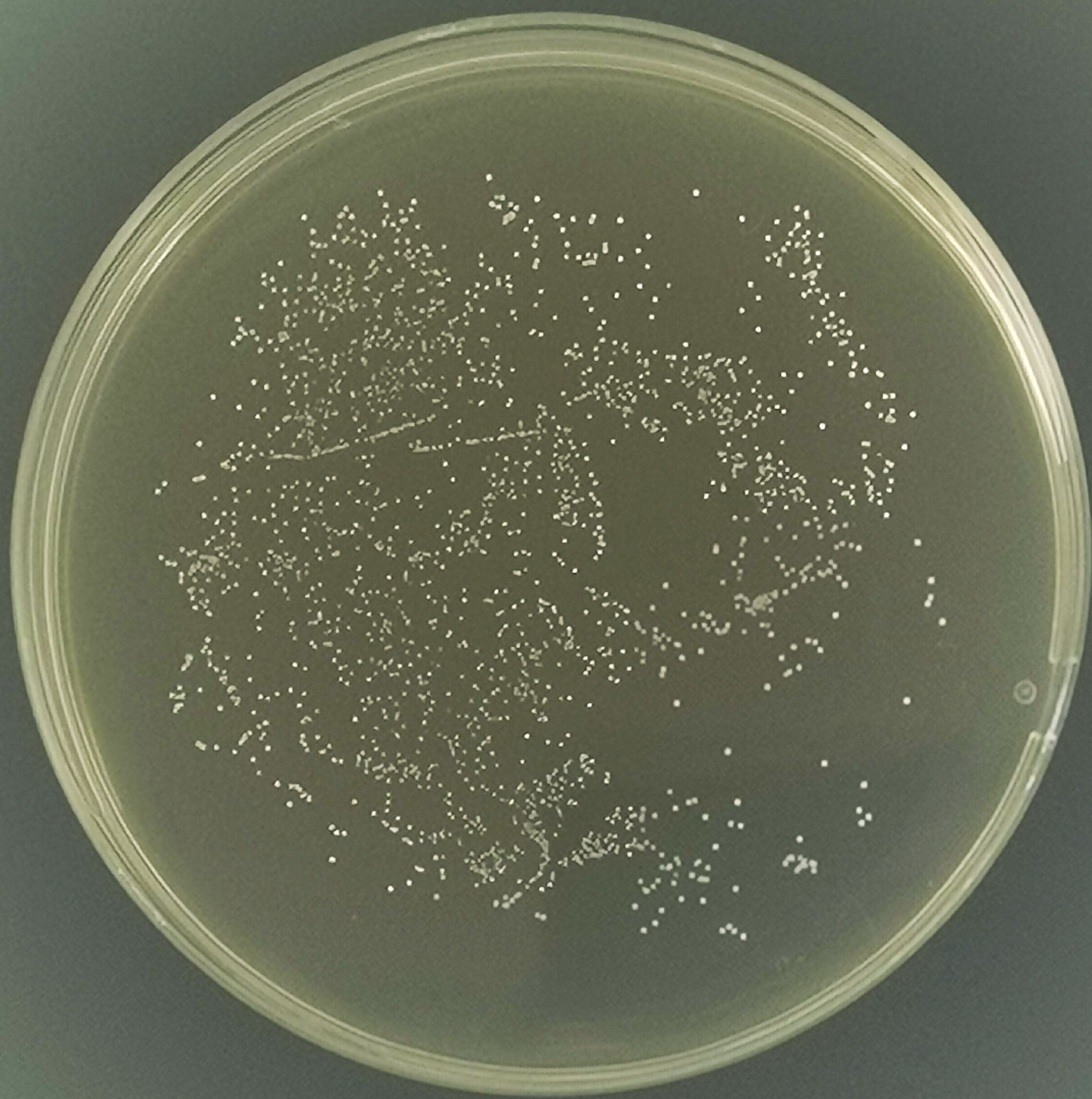

Supplement: S3 File — (ZIP) [file pone.0304143.s003.zip › S3 File.Antibacterial properties of the orthodontic adhesive/CFU/resin surface/0%.jpg]

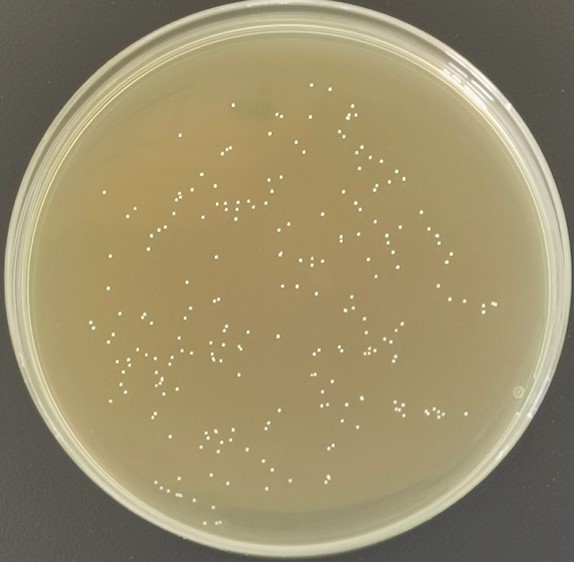

Supplement: S3 File — (ZIP) [file pone.0304143.s003.zip › S3 File.Antibacterial properties of the orthodontic adhesive/CFU/resin surface/1%.jpg]

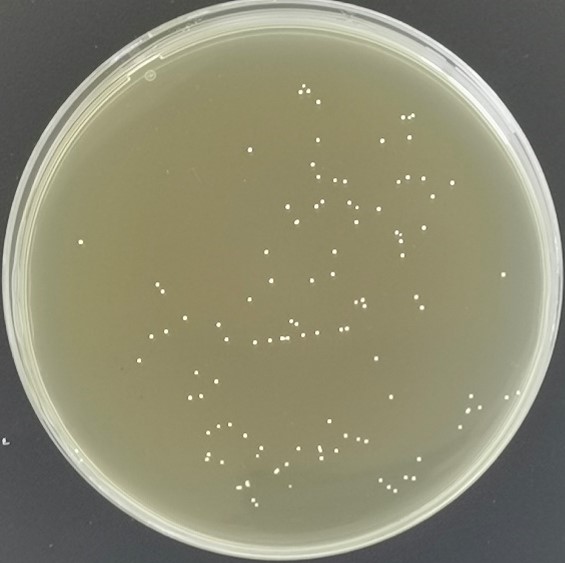

Supplement: S3 File — (ZIP) [file pone.0304143.s003.zip › S3 File.Antibacterial properties of the orthodontic adhesive/CFU/resin surface/3%.jpg]

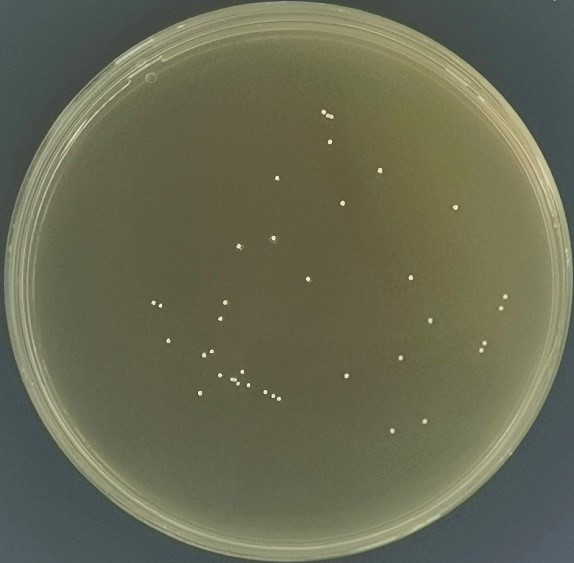

Supplement: S3 File — (ZIP) [file pone.0304143.s003.zip › S3 File.Antibacterial properties of the orthodontic adhesive/CFU/resin surface/5%.jpg]

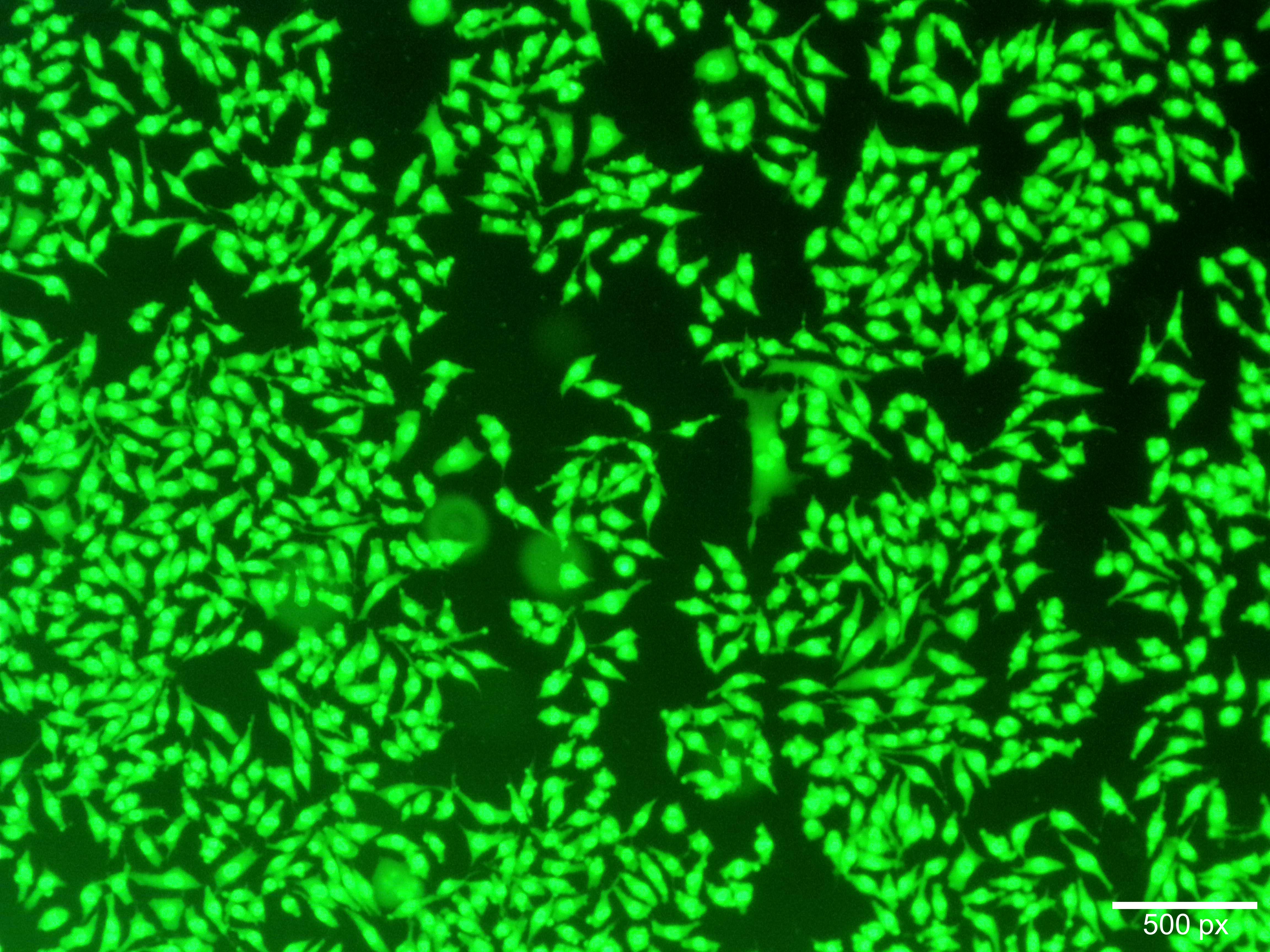

Supplement: S4 File — (ZIP) [file pone.0304143.s004.zip › S4 File.Biosafety performance of the orthodontic adhesive/AOEB/AO-1%.jpg]

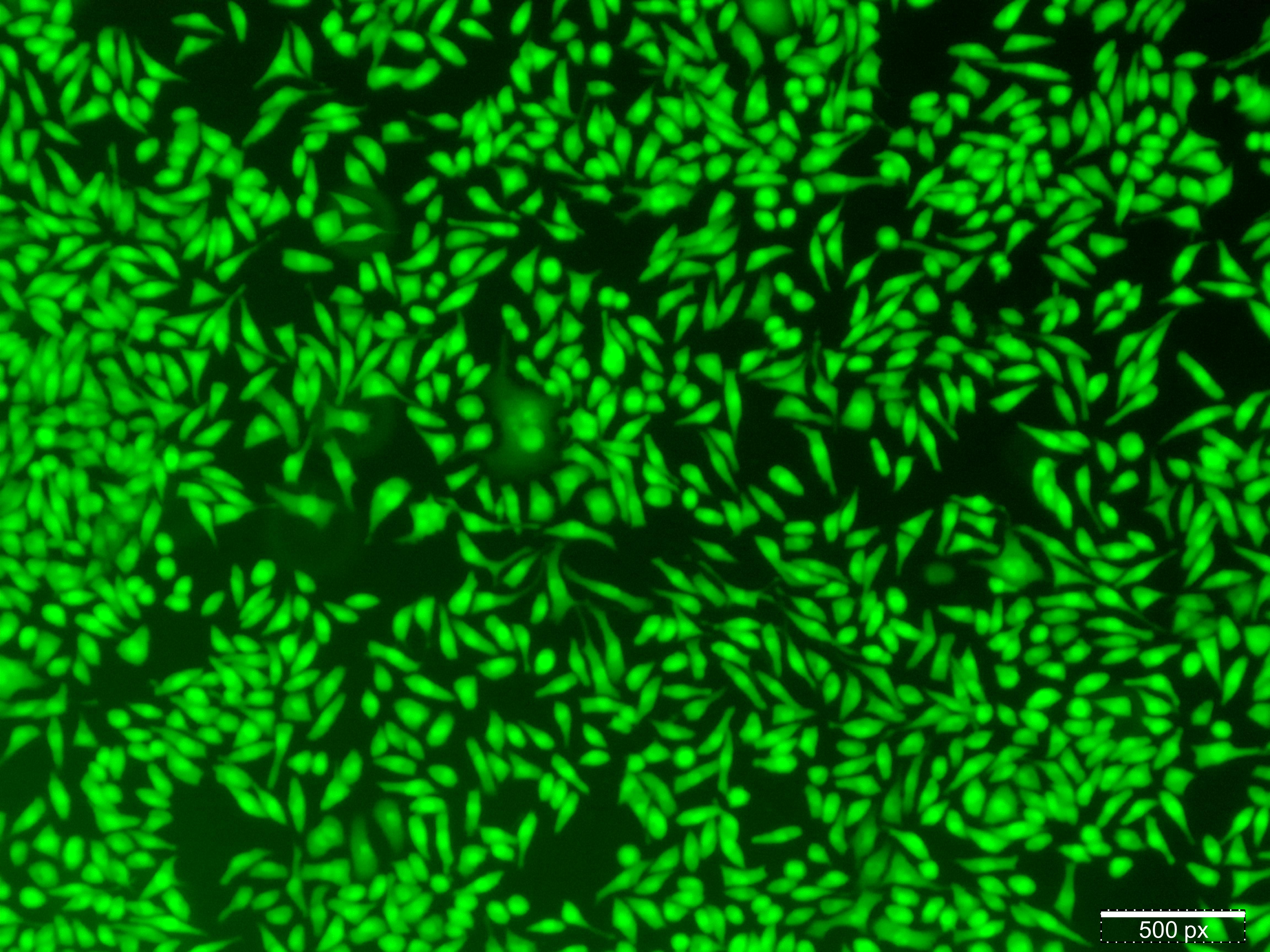

Supplement: S4 File — (ZIP) [file pone.0304143.s004.zip › S4 File.Biosafety performance of the orthodontic adhesive/AOEB/AO-3%.jpg]

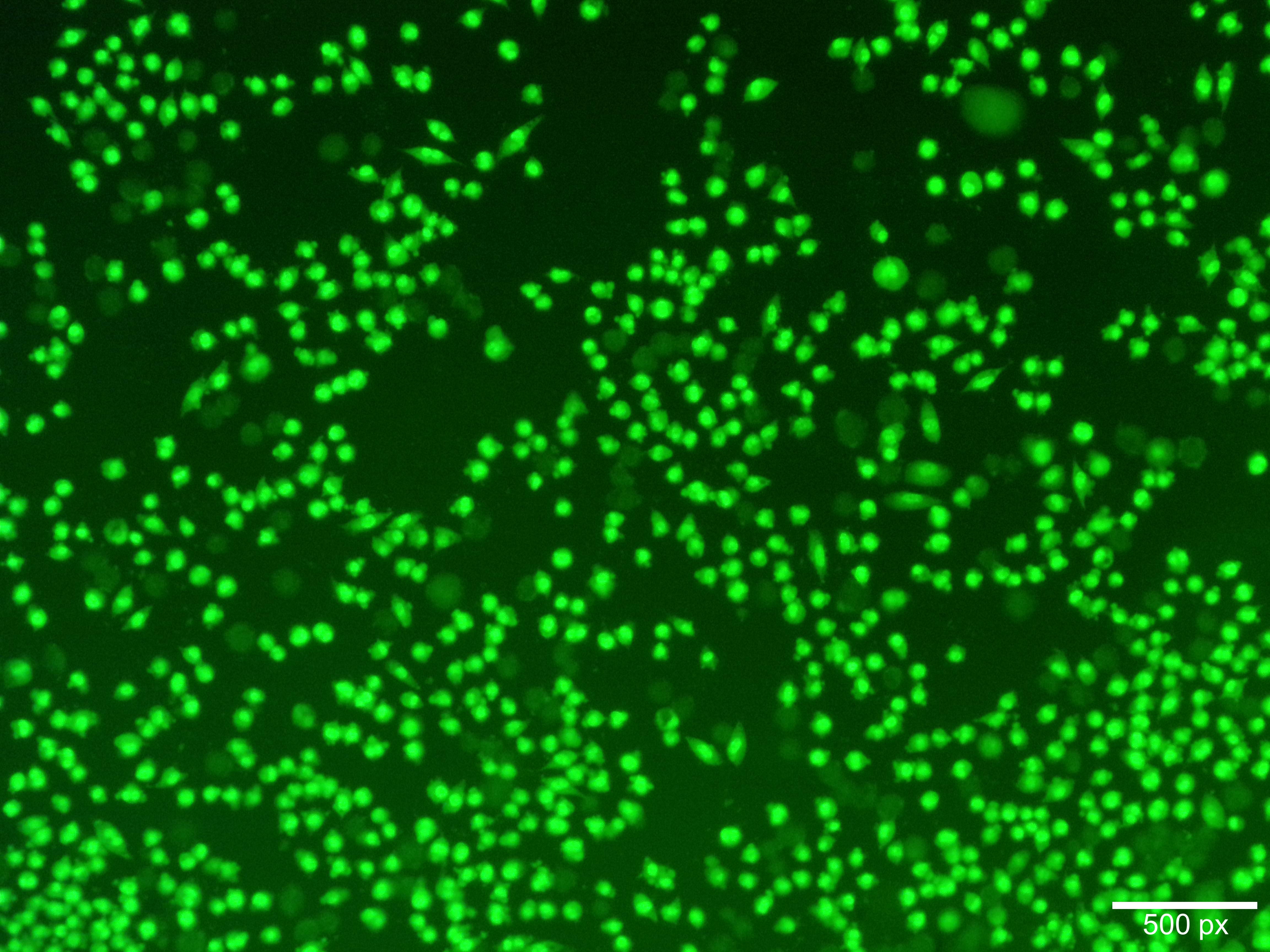

Supplement: S4 File — (ZIP) [file pone.0304143.s004.zip › S4 File.Biosafety performance of the orthodontic adhesive/AOEB/AO-5%.jpg]

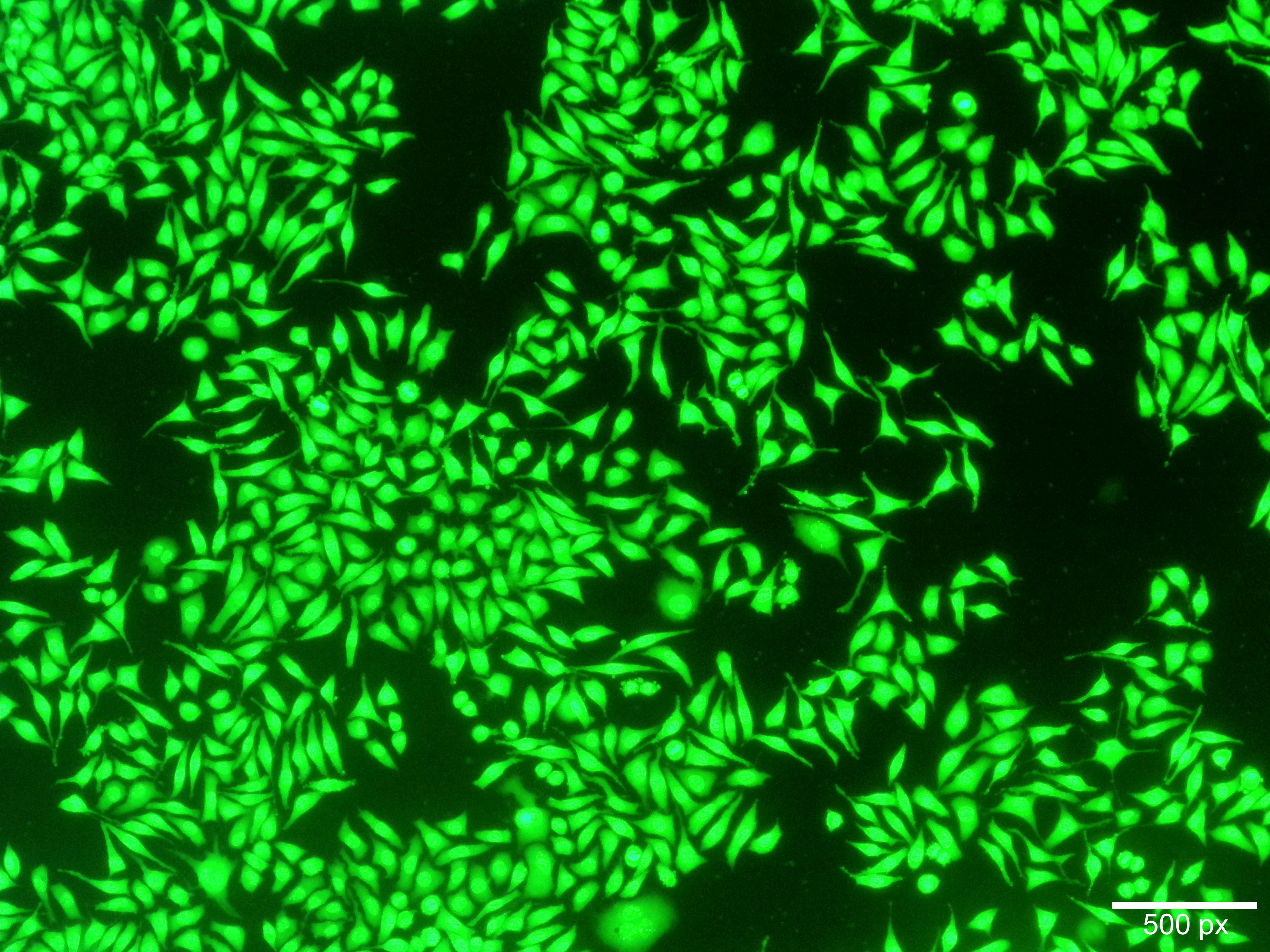

Supplement: S4 File — (ZIP) [file pone.0304143.s004.zip › S4 File.Biosafety performance of the orthodontic adhesive/AOEB/AO-Control.jpg]

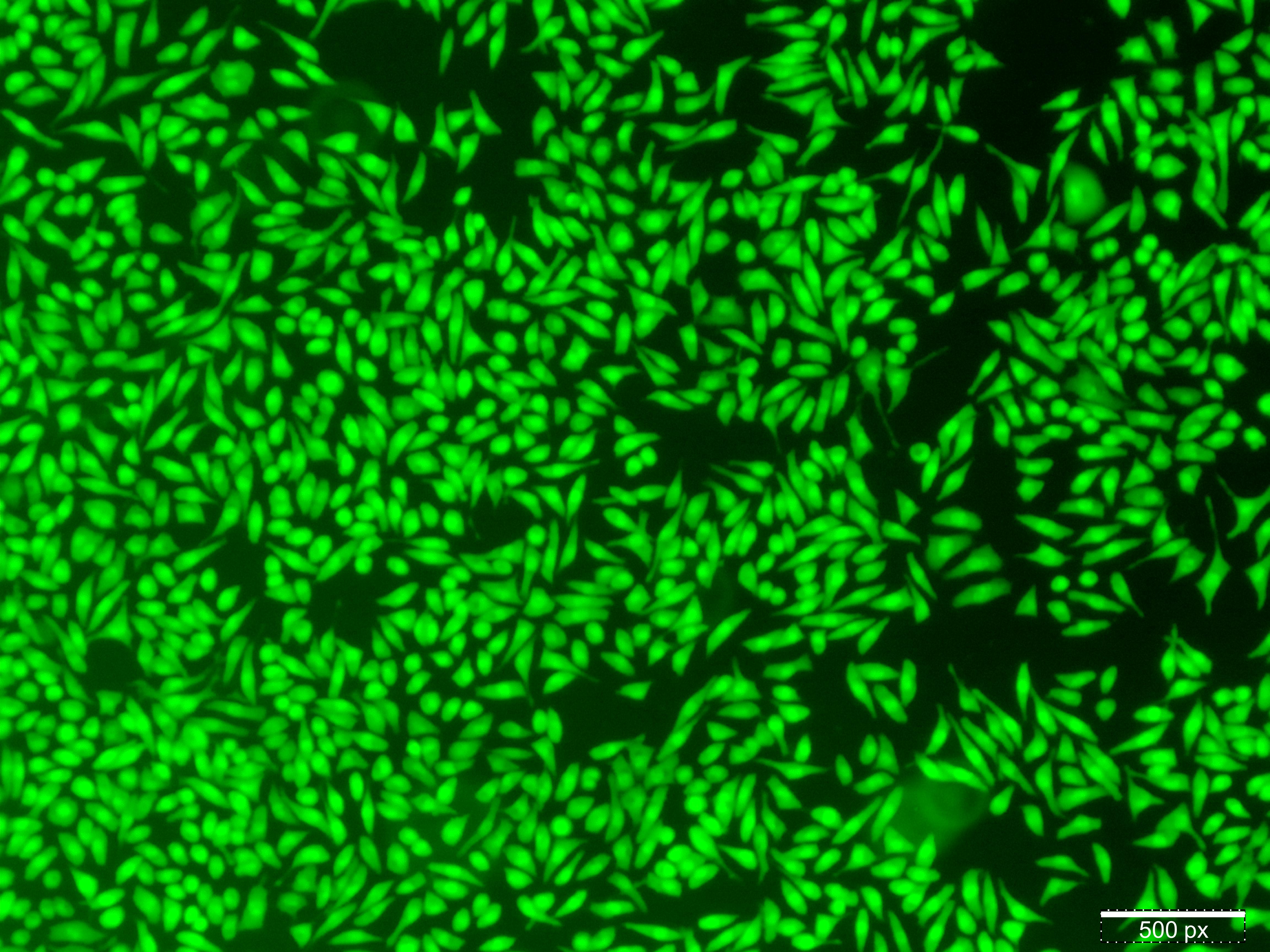

Supplement: S4 File — (ZIP) [file pone.0304143.s004.zip › S4 File.Biosafety performance of the orthodontic adhesive/AOEB/AO-Transbond XT.jpg]

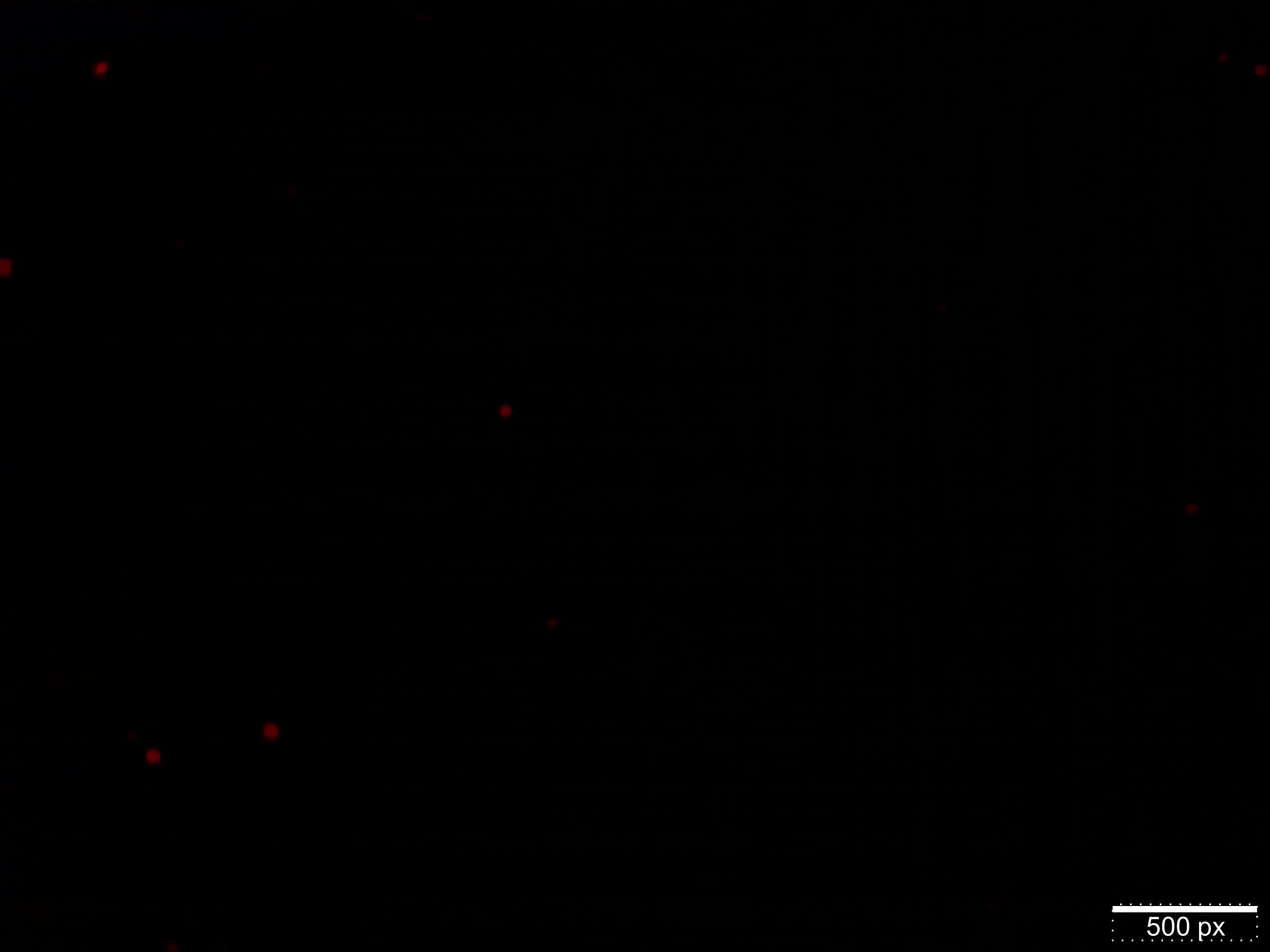

Supplement: S4 File — (ZIP) [file pone.0304143.s004.zip › S4 File.Biosafety performance of the orthodontic adhesive/AOEB/EB-1%.jpg]

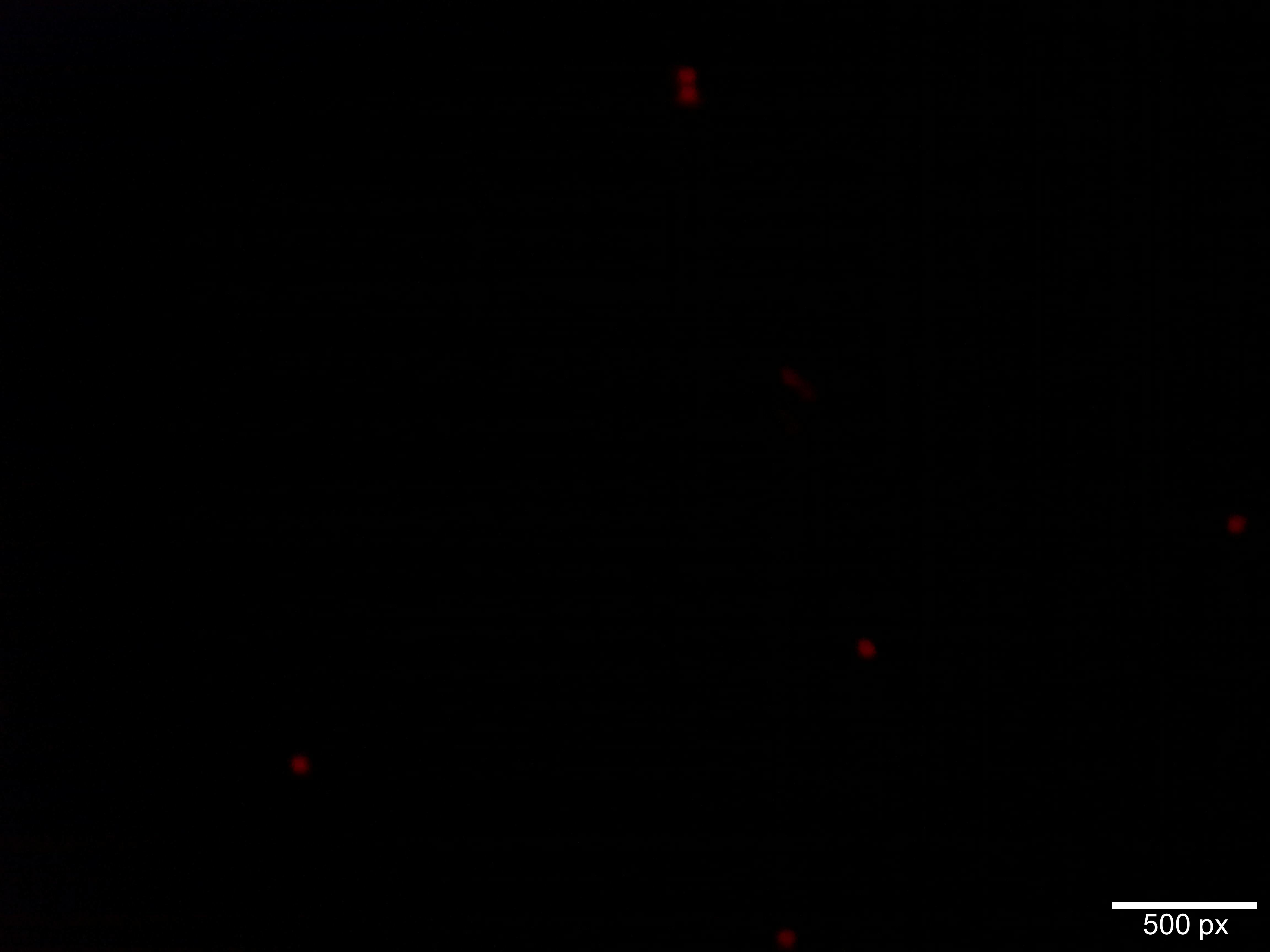

Supplement: S4 File — (ZIP) [file pone.0304143.s004.zip › S4 File.Biosafety performance of the orthodontic adhesive/AOEB/EB-3%.jpg]

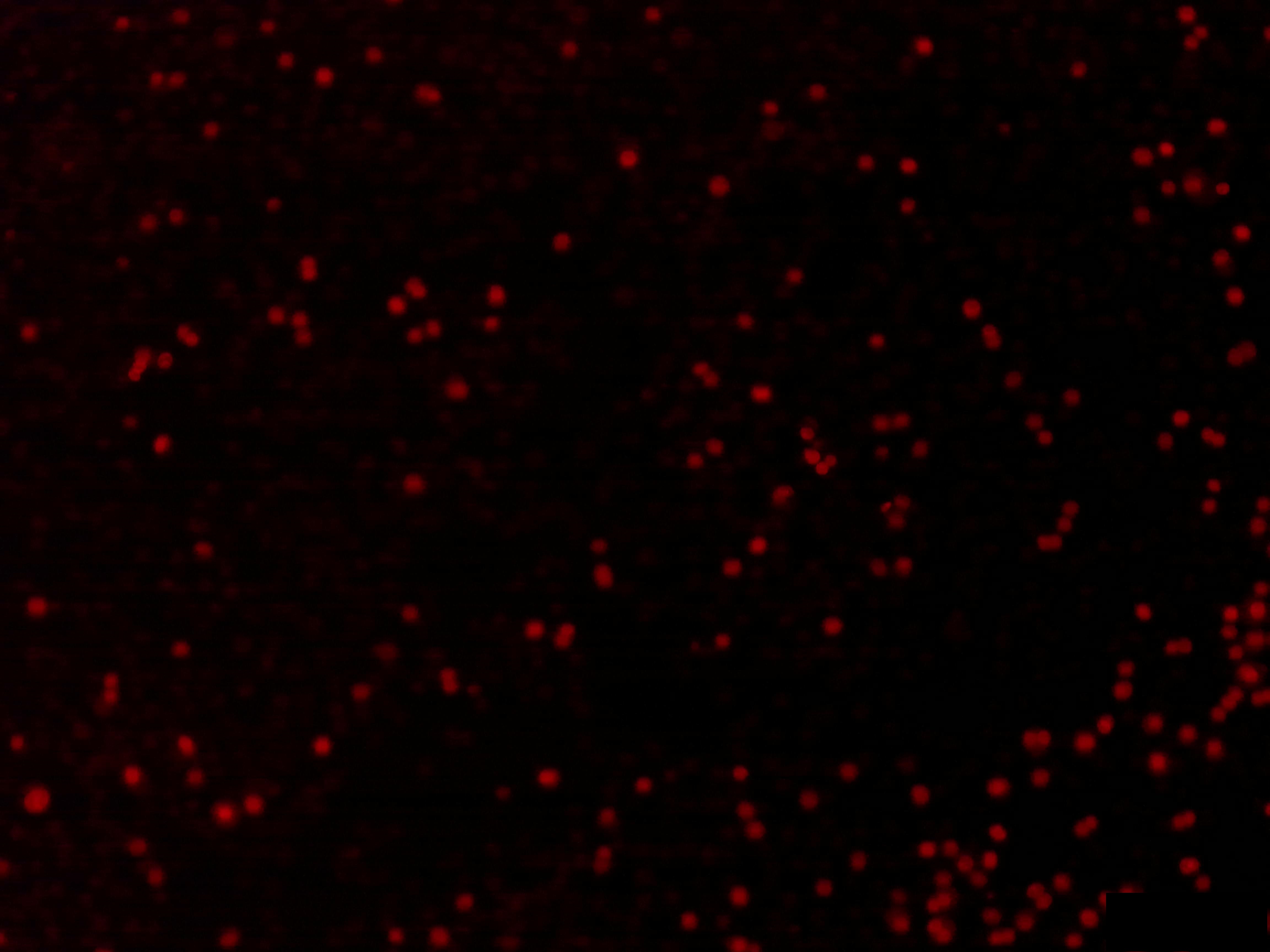

Supplement: S4 File — (ZIP) [file pone.0304143.s004.zip › S4 File.Biosafety performance of the orthodontic adhesive/AOEB/EB-5%.jpg]

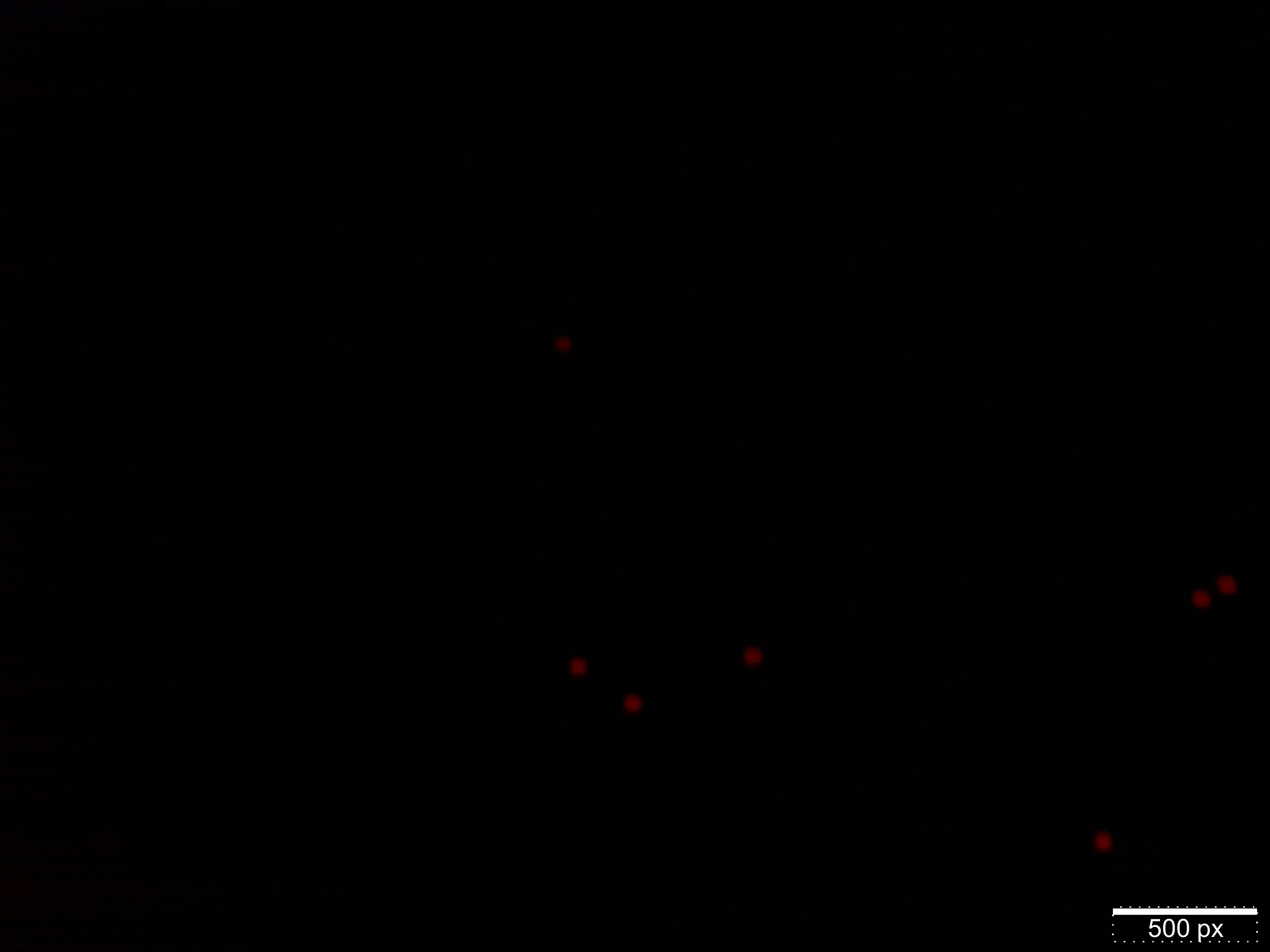

Supplement: S4 File — (ZIP) [file pone.0304143.s004.zip › S4 File.Biosafety performance of the orthodontic adhesive/AOEB/EB-Control.jpg]

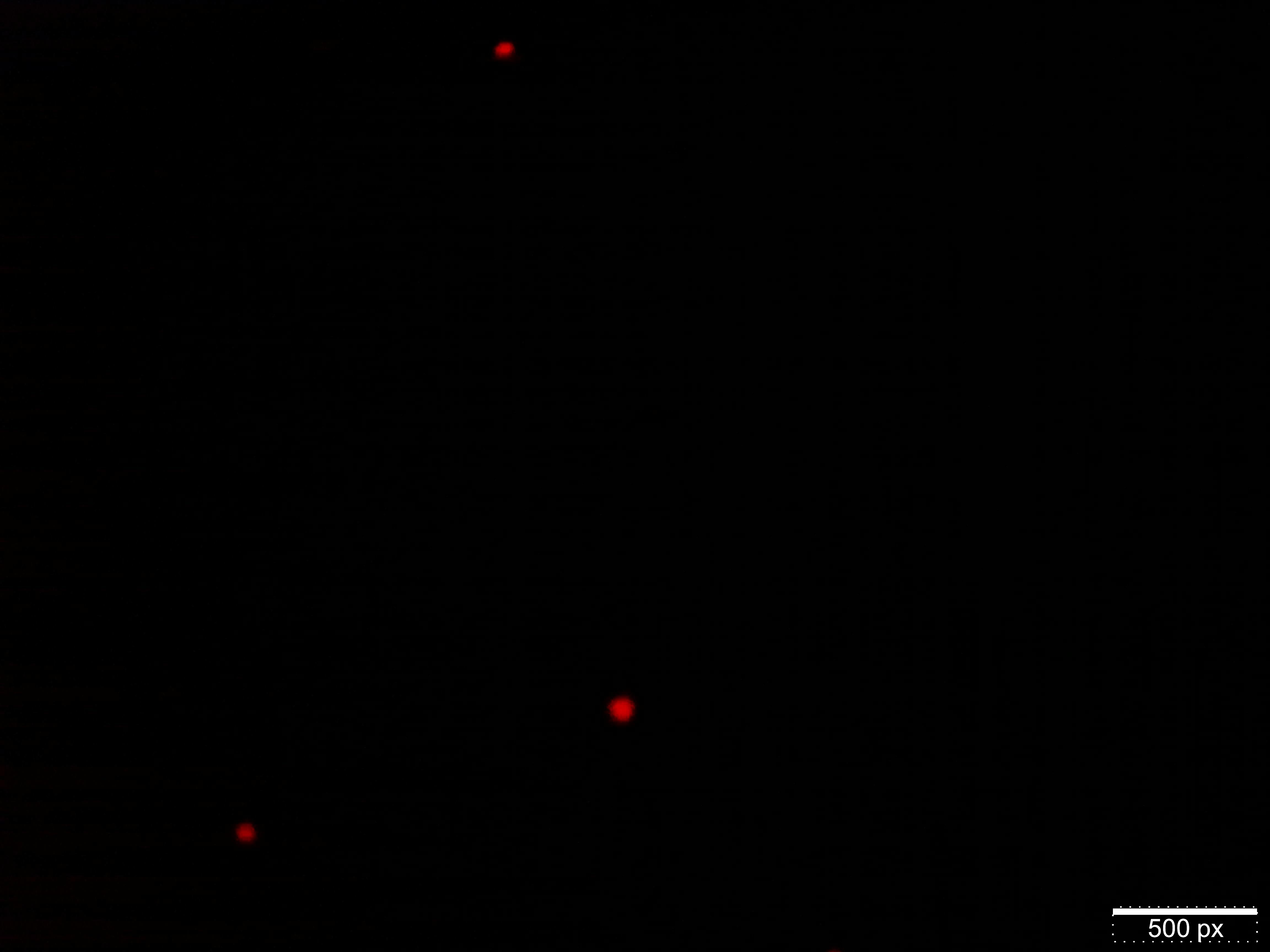

Supplement: S4 File — (ZIP) [file pone.0304143.s004.zip › S4 File.Biosafety performance of the orthodontic adhesive/AOEB/EB-Transbond xt.jpg]

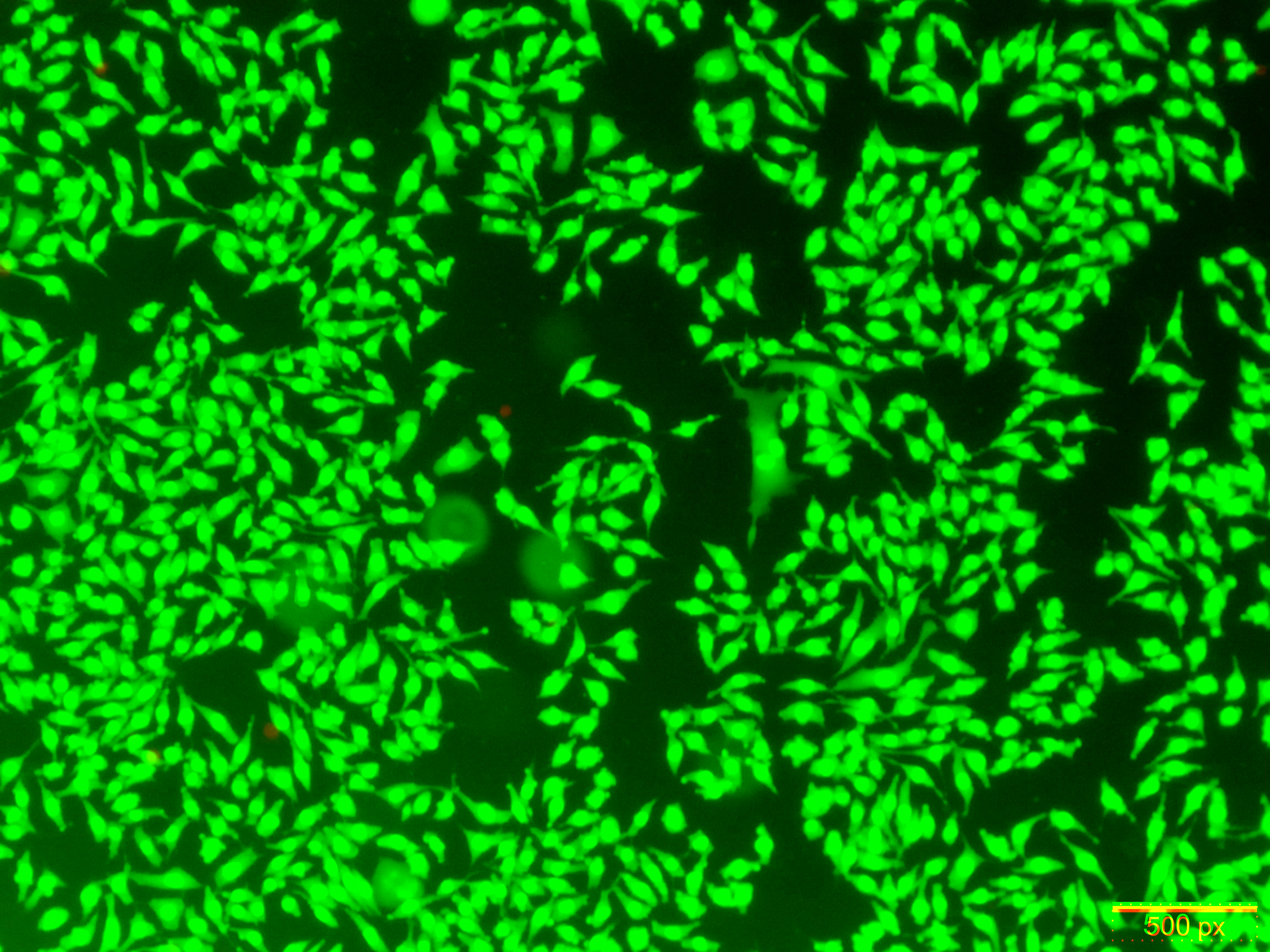

Supplement: S4 File — (ZIP) [file pone.0304143.s004.zip › S4 File.Biosafety performance of the orthodontic adhesive/AOEB/Merge-1%.tif]

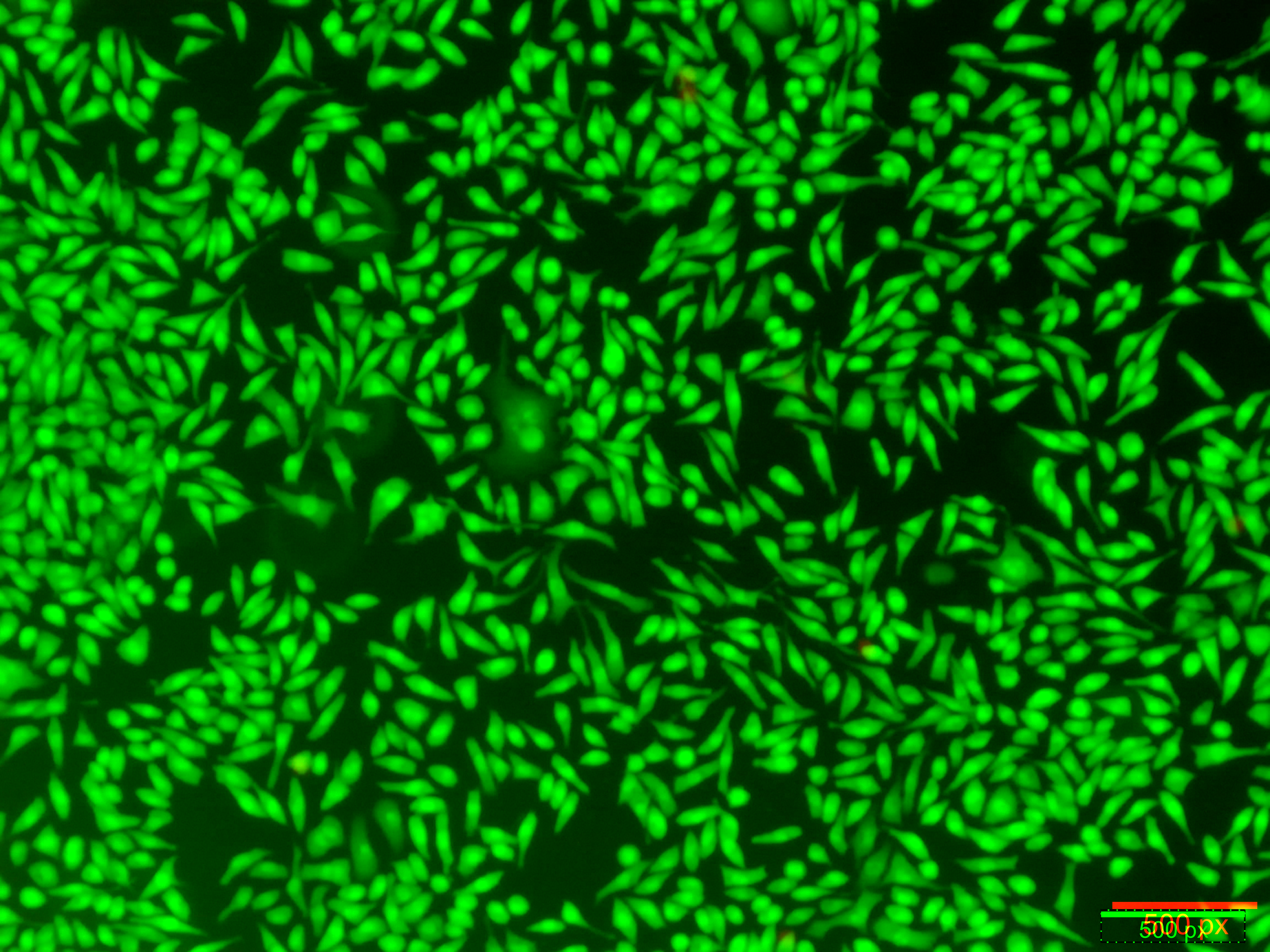

Supplement: S4 File — (ZIP) [file pone.0304143.s004.zip › S4 File.Biosafety performance of the orthodontic adhesive/AOEB/Merge-3%.tif]

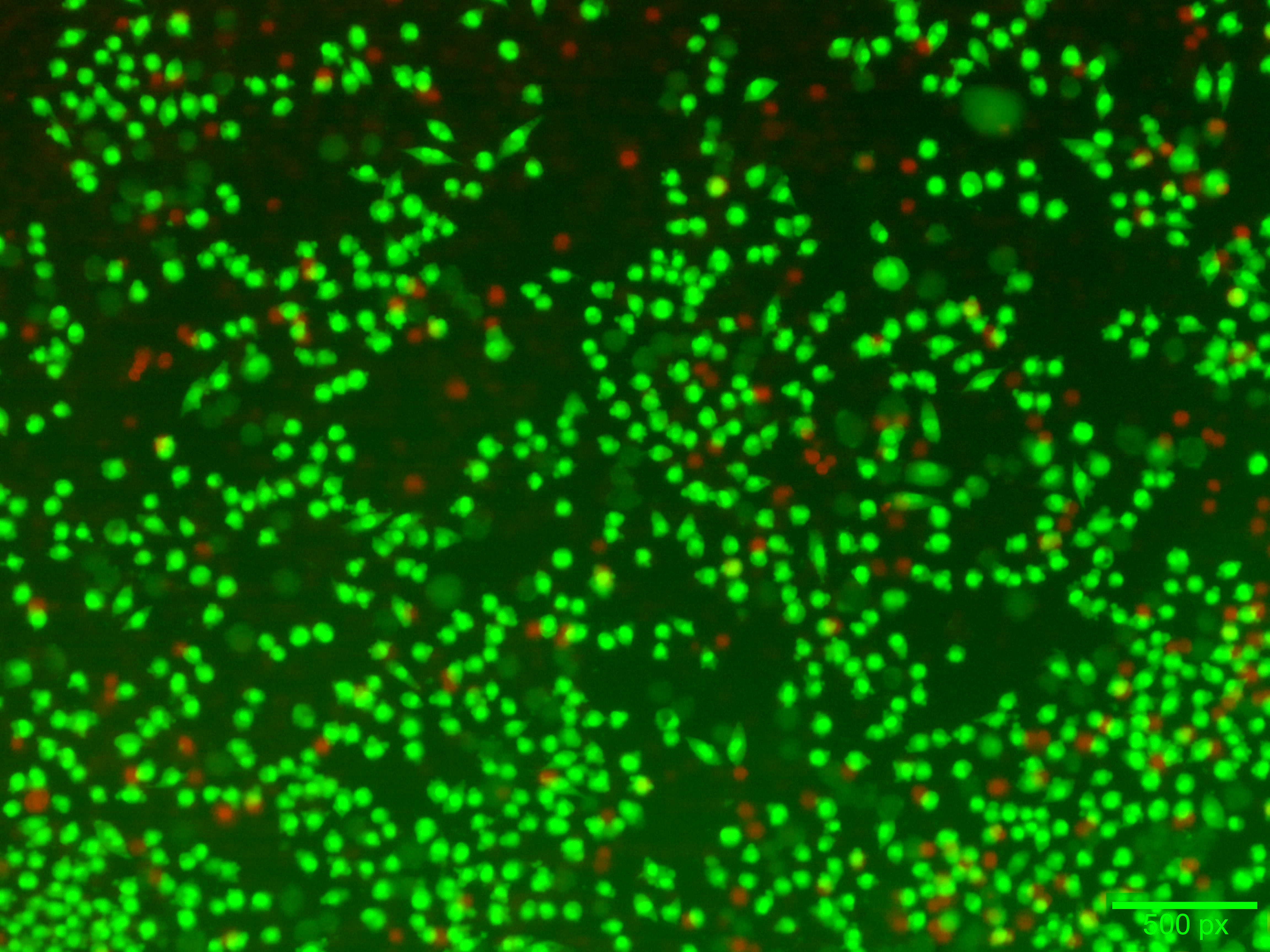

Supplement: S4 File — (ZIP) [file pone.0304143.s004.zip › S4 File.Biosafety performance of the orthodontic adhesive/AOEB/Merge-5%.tif]

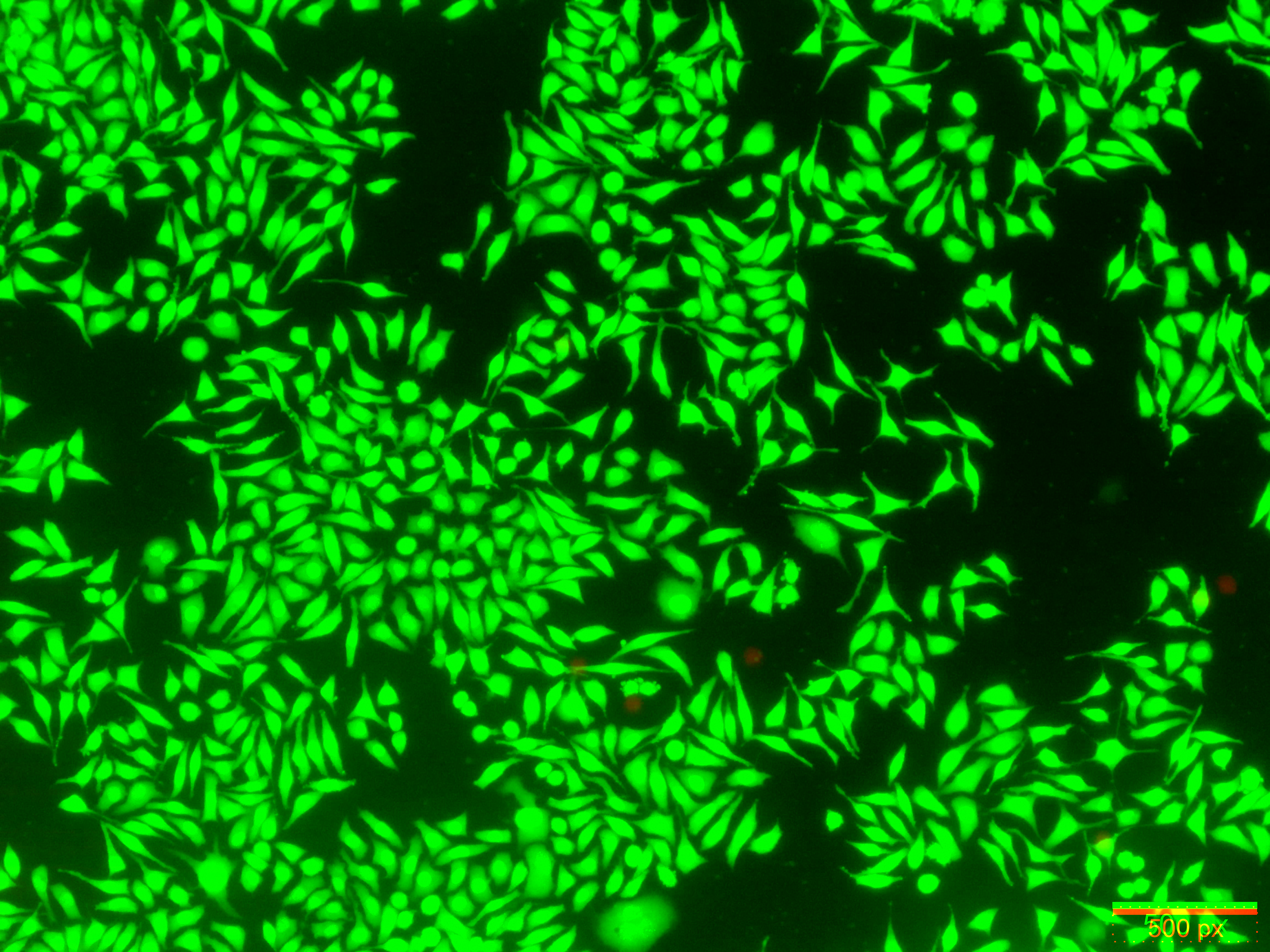

Supplement: S4 File — (ZIP) [file pone.0304143.s004.zip › S4 File.Biosafety performance of the orthodontic adhesive/AOEB/Merge-Control.tif]

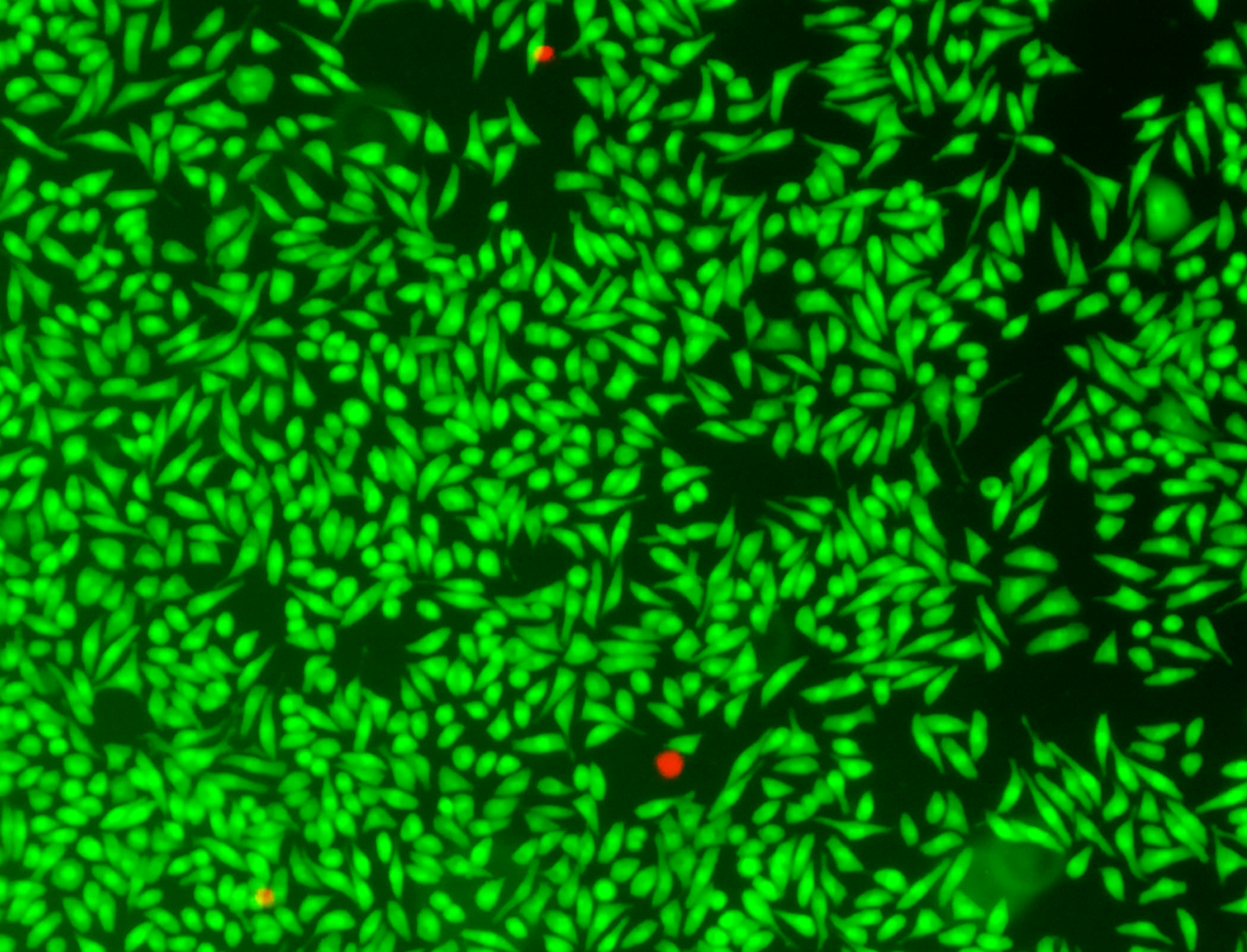

Supplement: S4 File — (ZIP) [file pone.0304143.s004.zip › S4 File.Biosafety performance of the orthodontic adhesive/AOEB/Merge-Transbond XT.tif]

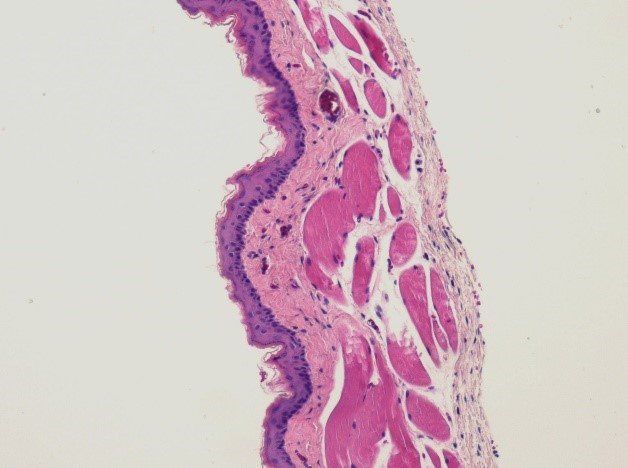

Supplement: S4 File — (ZIP) [file pone.0304143.s004.zip › S4 File.Biosafety performance of the orthodontic adhesive/HE/Control groupX100.jpg]

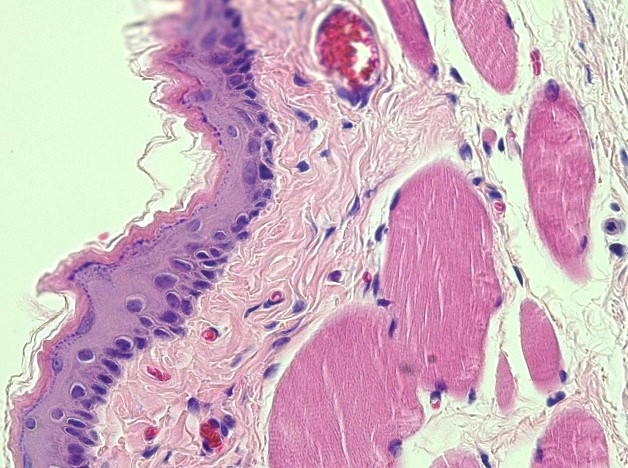

Supplement: S4 File — (ZIP) [file pone.0304143.s004.zip › S4 File.Biosafety performance of the orthodontic adhesive/HE/Control groupX400.jpg]

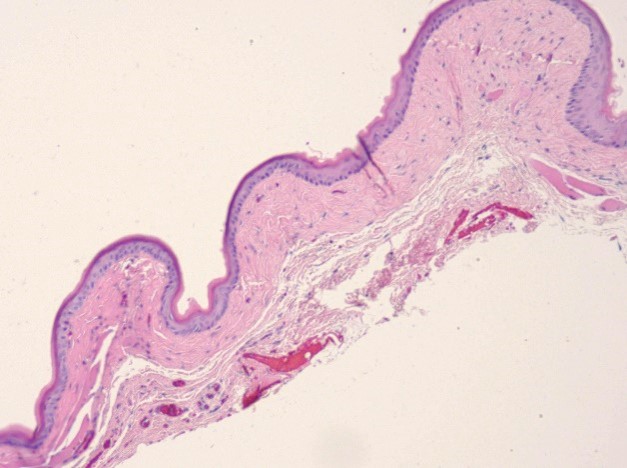

Supplement: S4 File — (ZIP) [file pone.0304143.s004.zip › S4 File.Biosafety performance of the orthodontic adhesive/HE/Experimental group X100.jpg]

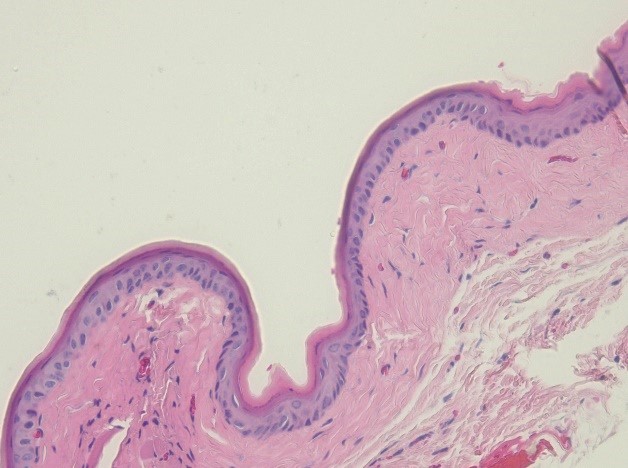

Supplement: S4 File — (ZIP) [file pone.0304143.s004.zip › S4 File.Biosafety performance of the orthodontic adhesive/HE/Experimental group X400.jpg]
